# First report of the genus *Bicorniphontodes* (Copepoda, Harpacticoida, Ancorabolidae) in South Korea, with descriptions of three new species

**DOI:** 10.7717/peerj.12530

**Published:** 2021-12-07

**Authors:** Jong Guk Kim, Jimin Lee, Kyuhee Cho

**Affiliations:** Marine Ecosystem Research Center, Korea Institute of Ocean Science and Technology, Busan, Republic of Korea

**Keywords:** Cladistic analysis, Crustacea, East Asia, Morphology, Taxonomy

## Abstract

We report the occurrence of the genus *Bicorniphontodes* George, Glatzel & Schröder, 2019 in Korean waters, with descriptions of three new species: *Bicorniphontodes lacuna* sp. nov., *B*. *comptus* sp. nov., and *B*. *huysi* sp. nov. Morphology analysis was carried out to reconstruct the phylogenetic relationships of *Bicorniphontodes* species, including the three new species. Parsimony analysis based on 41 characters revealed that the three new species are clustered together as a monophyletic clade, of which *B*. *horstgeorgei* (George & Gheerardyn, 2015) is a sister species. The monophyletic status of three new species was supported by five synapomorphies, such as the micro-morphological conditions of the exopodal segments of the first leg, coxae of the second to fourth legs, exopod of the fifth leg in the female and baseoendopod of the fifth leg in the male, and the maxillular endopod represented by two setae. These three species can be easily distinguished based on the morphology of the rostrum, cephalothoracic processes, female genital double-somite, caudal rami, and second endopodal segment of the third leg in the male. The taxonomic position of *B*. *bicornis sensu* Kim, 2013 in Korean fauna was reevaluated based on the newly collected material from Udo Islet near Jeju Island; this resulted in a synonym of *B*. *huysi* sp. nov.

## Introduction

Since its establishment, the family Ancorabolidae Sars, 1909 has been modified and revised (Lee & Huys, 2019; George, 2020). Systematic confusion began with the incorporation of the genus *Laophontodes* Scott T., 1894 into the family by Lang (1936), resulting in an extension of the familial boundary. Subsequently, Lang (1944, 1948) subdivided the family into two subfamilies, Ancorabolinae Sars, 1909 and Laophontodinae Lang, 1944; the latter subfamily was erected for *Laophontodes* based on plesiomorphic characters such as endopod prehensility in the first leg, basis elongation in the first leg, and the presence of the antennary exopod. However, the subfamily was not characterized by any autapomorphies (George, 2006; George, 2017); thus, some researchers began to question Lang’s subdivision of Ancorabolidae and Laophontodinae (*e.g*., Gee & Fleeger, 1986; Conroy-Dalton, 2004; George, 2006), and attempted to resolve this ambiguous phylogenetic relationship (Conroy-Dalton & Huys, 2000; Conroy-Dalton, 2001; Gheerardyn & George, 2010; Gheerardyn & Lee, 2012). Recently, George (2020) introduced a new phylogeny for the family and its subfamilies, based on a careful comparison of morphological characters: the *Ceratonotus*-group *sensu* Conroy-Dalton, 2001 (Ancorabolinae) was reassigned to the family Cletodidae Scott T., 1904; the remaining *Ancorabolus*-group *sensu* Conroy-Dalton & Huys, 2000 was elevated to subfamily rank; and the assignment of the genus *Ancorabolina* George, 2006 from Ancorabolinae to Laophontodinae suggested by Lee & Huys (2019) was formally adopted.

Revisions of *Laophontodes* revealed its polyphyletic status and led to the establishment of several new genera, such as *Bicorniphontodes* George, Glatzel & Schröder, 2019, *Calypsophontodes* Gheerardyn & Lee, 2012, *Lobopleura* Conroy-Dalton, 2004, *Paralaophontodes* Lang, 1965, and *Rostrophontodes* Lee & Huys, 2019 (Conroy-Dalton, 2004; Gheerardyn & Lee, 2012; George, Glatzel & Schröder, 2019; Lee & Huys, 2019). The genus *Bicorniphontodes* was established on the basis of six synapomorphies, including the presence of mediolateral extensions and posterolateral corniform processes on the cephalothorax. This genus currently comprises five species: *Bicorniphontodes bicornis* (Scott A., 1896), *B*. *clarae* George, Glatzel & Schröder, 2019, *B*. *hamatus* (Thomson, 1883), *B*. *horstgeorgei* (George & Gheerardyn, 2015), and *B*. *ornatus* (Krishnaswamy, 1957). They occur from shallow to littoral waters (George, Glatzel & Schröder, 2019). In Korean waters, Kim (2013) reported *B*. *bicornis* from Seoqwipo at Jeju Island, but its taxonomic status is unclear (Lee & Huys, 2019; Song et al., 2020).

During our ongoing research on Korean marine harpacticoids, we found two unknown *Bicorniphontodes* species from the East Sea and Jeju Strait, as well as specimens of *B*. *bicornis sensu* Kim, 2013 from Udo Islet (a small island located northeast of Jeju Island). Here, we provide detailed morphological descriptions of these three species to shed light on the diversity of the genus in Korean waters, and to clarify the taxonomic position of *B*. *bicornis sensu* Kim, 2013.

## Materials & methods

**Figure 1:**
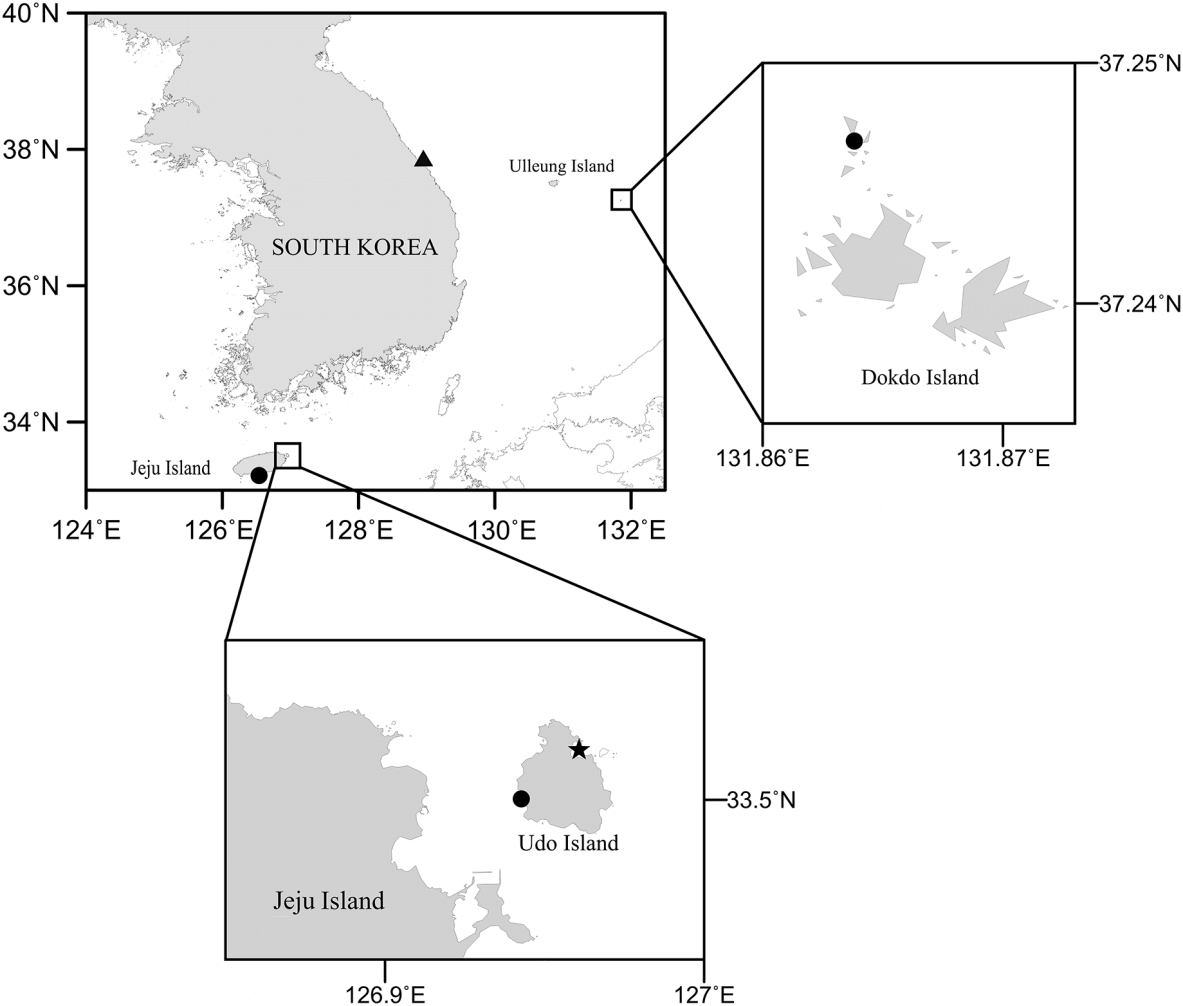
Map of the study area collected three new *Bicorniphontodes* species. Filled circle, *B*. *lacuna* sp. nov., filled triangle, *B*. *comptus* sp. nov., filled star, *B*. *huysi* sp. nov.

To investigate the diversity of Korean harpacticoids in sediments, field campaigns were carried out along the coastlines and on islands using hand nets and Smith-McIntyre grabs, and by SCUBA diving. Sediment samples were immediately fixed with a 5% formaldehyde solution. In the laboratory, meiofaunal animals were extracted by the decanting method (Pfannkuche & Thiel, 1988) with a sieve (mesh size, 50 µm) and stored in 95% ethanol. Subsequently, harpacticoids were sorted under a stereomicroscope (M165C; Leica, Germany) and *Bicorniphontodes* species were temporarily placed in lactic acid. Morphological examinations were performed using a differential contrast microscope (BX53; Olympus, Tokyo, Japan) equipped with a drawing tube. Pencil drawings of habitus and appendages were made using the sandwich mounting method (Huys & Boxshall, 1991) and the hanging drop method (Humes & Gooding, 1964), respectively. Dissected parts of the examined specimens were mounted in lactophenol and covered with a coverslip (to prevent urosome depression, a small amount of paraffin was added in the mounting media); they were then sealed with Canada balsam. Digital inking was done using a Wacom drawing tablet (Cintiq DTK-1301; Wacom, Saitama, China) running Adobe Photoshop CS6 software (Adobe Inc., San Jose, CA, USA). A map showing the sampling stations for *Bicorniphontodes* species (Fig. 1) was generated using Surfer software (version 12). Scale bars are in micrometers. The examined material was deposited in the collection of the Marine Biodiversity Institute of Korea (MABIK), Seocheon, Korea and the Marine Interstitial fauna Resources Bank (MInRB) in the Korea Institute of Ocean Science and Technology (KIOST), Busan, Korea.

The morphological terminology follows Huys & Boxshall (1991). Abbreviations used in the text are as follows: enp-1(-2, -3) = proximal (middle, distal) segment of the endopod; exp-1(-2, -3) = proximal (middle, distal) segment of the exopod; P1–P6 = first to sixth thoracic leg.

A cladistic analysis was conducted to determine possible phylogenetic relationships of the *Bicorniphontodes* species, including three new species described herein. Unfortunately, *B*. *hamatus* and *B*. *ornatus* were excluded from the ingroup because their original descriptions are insufficient for a detailed assessment of morphological characters. The cladogram was rooted using an outgroup taxon, *Laophontodes typicus* Scott T., 1894, known to be closely related to *Bicorniphontodes* (George, 2020). The character matrix was coded from 41 selected morphological characters as binary (0–1) or multistate (0–n). All characters were coded in accordance with the principle of “oligomerization” (the fusion of segments or loss of setal elements on appendages), which is considered an irreversible evolutional process in copepods (Huys & Boxshall, 1991). A presumably plesiomorphic state was represented by “0”, a presumably apomorphic state by “1” or “2”, and missing data by “?”. Parsimony analysis was performed using the program NONA (Goloboff, 1999) interfaced with the program WinClada software (ver. 1.00.08) (Nixon, 2002). Ratchet Island Hopper analysis (Nixon, 1999) was conducted with the following settings: 1,000 interations, five trees to hold/interation, 10 characters to sample, “amb-poly=”, 10 random constraint level, and 0 random seed. Bootstrap values were also computed using the Tree-Bisection-Reconnection (TBR) method in WinClada software with the following options: 500 replications, 10 replicate searches, one starting tree per replication, and a maximum of 100 trees. Within the selected cladograms, apomorphic and homoplastic characters were arranged using the “Unambig changes only” option.

The electronic version of this article in Portable Document Format (PDF) will represent a published work according to the International Commission on Zoological Nomenclature (ICZN), and hence the new names contained in the electronic version are effectively published under that Code from the electronic edition alone. This published work and the nomenclatural acts it contains have been registered in ZooBank, the online registration system for the ICZN. The ZooBank LSIDs (Life Science Identifiers) can be resolved and the associated information viewed through any standard web browser by appending the LSID to the prefix http://zoobank.org/. The LSID for this publication is: [urn:lsid:zoobank.org:pub:CA40EAE8-2B46-40A1-88D5-B32A80ADDD05]. The online version of this work is archived and available from the following digital repositories: PeerJ, PubMed Central and CLOCKSS.

## Results

**Taxonomy**

Phylum Arthropoda von Siebold, 1848

Subphylum Crustacea Brünnich, 1772

Superclass Multicrustacea Regier, Shultz, Zwick, Hussey, Ball, Wetzer, Martin & Cunningham, 2010

Subclass Copepoda Milne-Edwards, 1840

Order Harpacticoida Sars G.O., 1903

Family Ancorabolidae Sars G.O., 1909

Genus *Bicorniphontodes* George, Glatzel & Schröder, 2019

*Bicorniphontodes lacuna* sp. nov.

urn:lsid:zoobank.org:act:CEC926FC-DC5C-4E2A-82E2-A27DEEC37173

Figures 2–8

**Figure 2:**
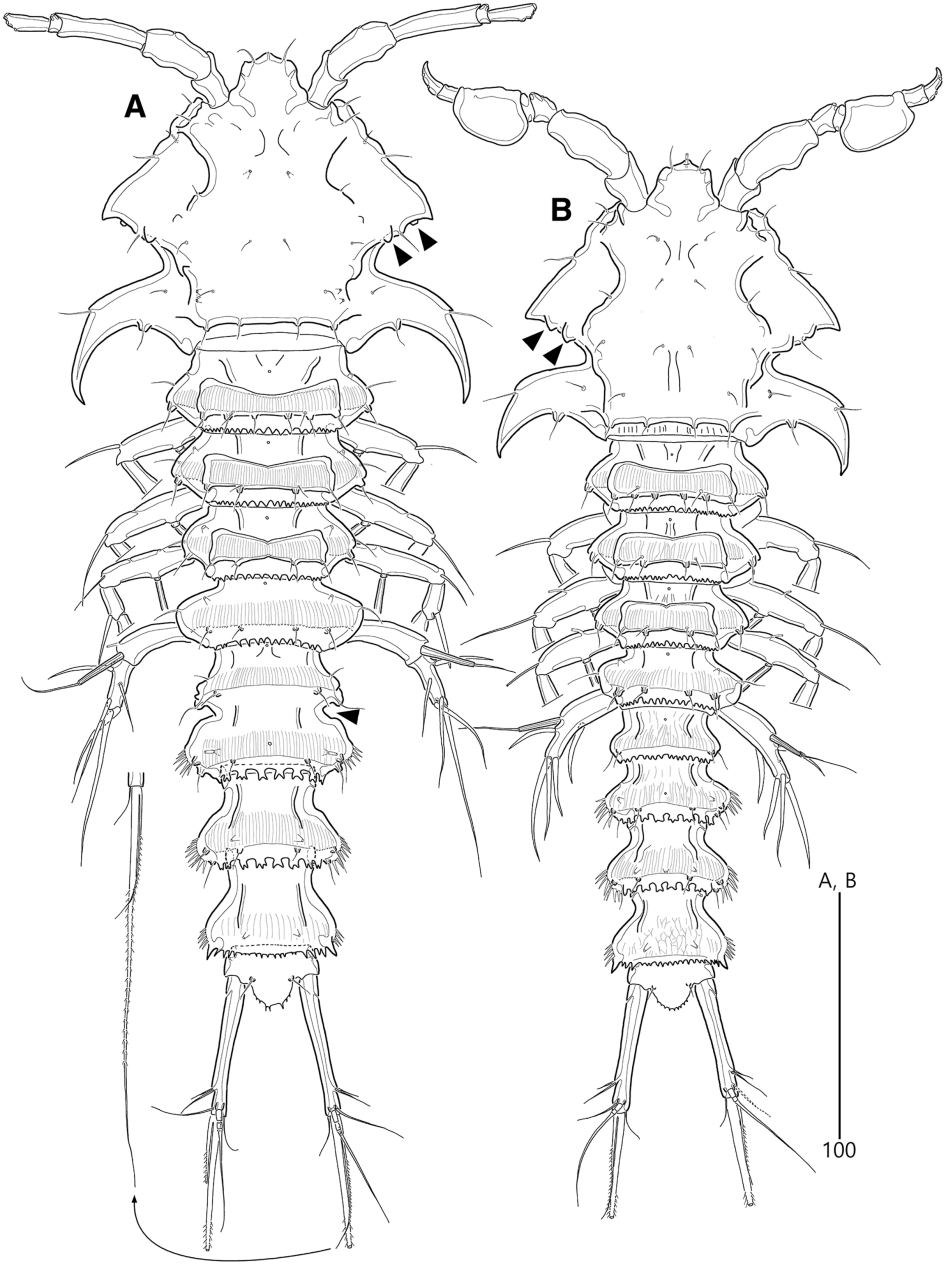
*Bicorniphontodes lacuna* sp. nov., female, holotype (A), male, paratype 1 (B). (A) Female habitus, dorsal; (B) Male habitus, dorsal. Arrowheads indicate apomorphies of *B*. *lacuna* sp. nov.

**Figure 3:**
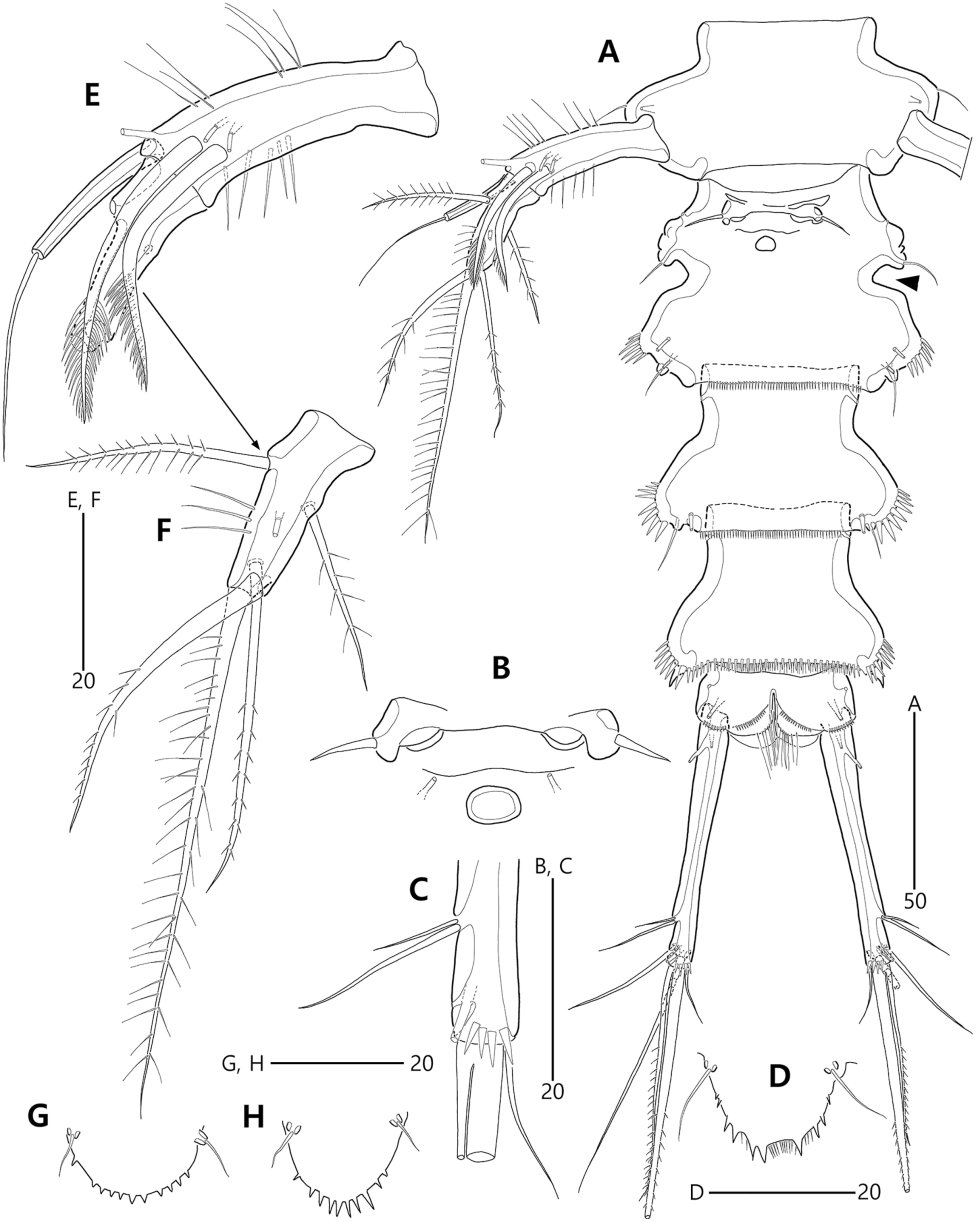
*Bicorniphontodes lacuna* sp. nov., female, holotype (A–F), paratype 2 (G), paratype 6 in a vial with other paratypes (H). (A) Urosome, ventral; (B) Genital field; (C) Distal part of cauda ramus, ventral; (D) Anal operculum, dorsal; (E) P5 baseoendopod and exopod omitted its setal armature, ventral; (F) P5 exopod, ventral; (G, H) Various shapes of anal operculum, dorsal. Arrowhead indicates an apomorphy of *B*. *lacuna* sp. nov.

**Figure 4:**
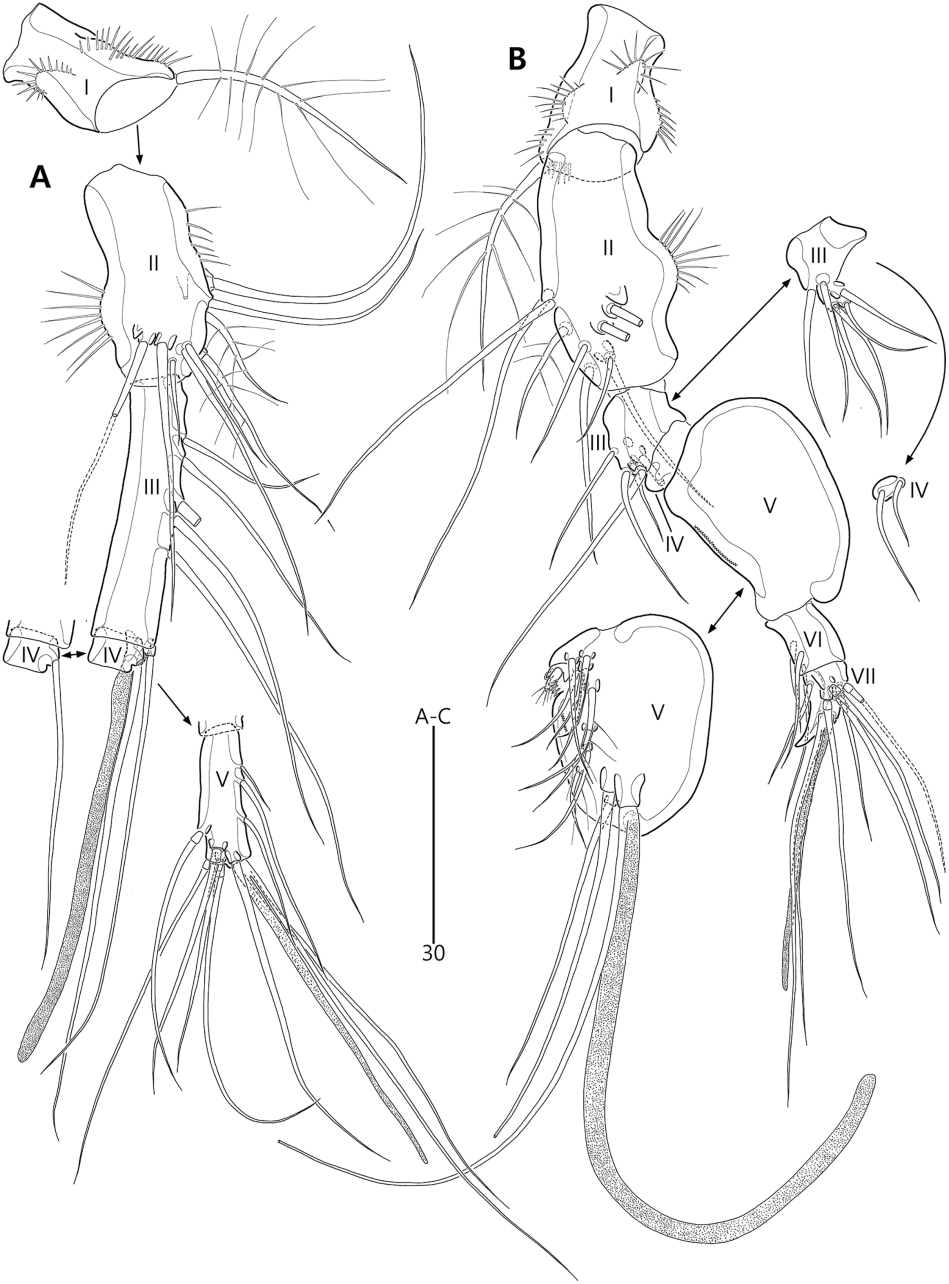
*Bicorniphontodes lacuna* sp. nov., female, holotype (A), male, paratype 1 (B). (A) Female antennule; (B) Male antennule.

**Figure 5:**
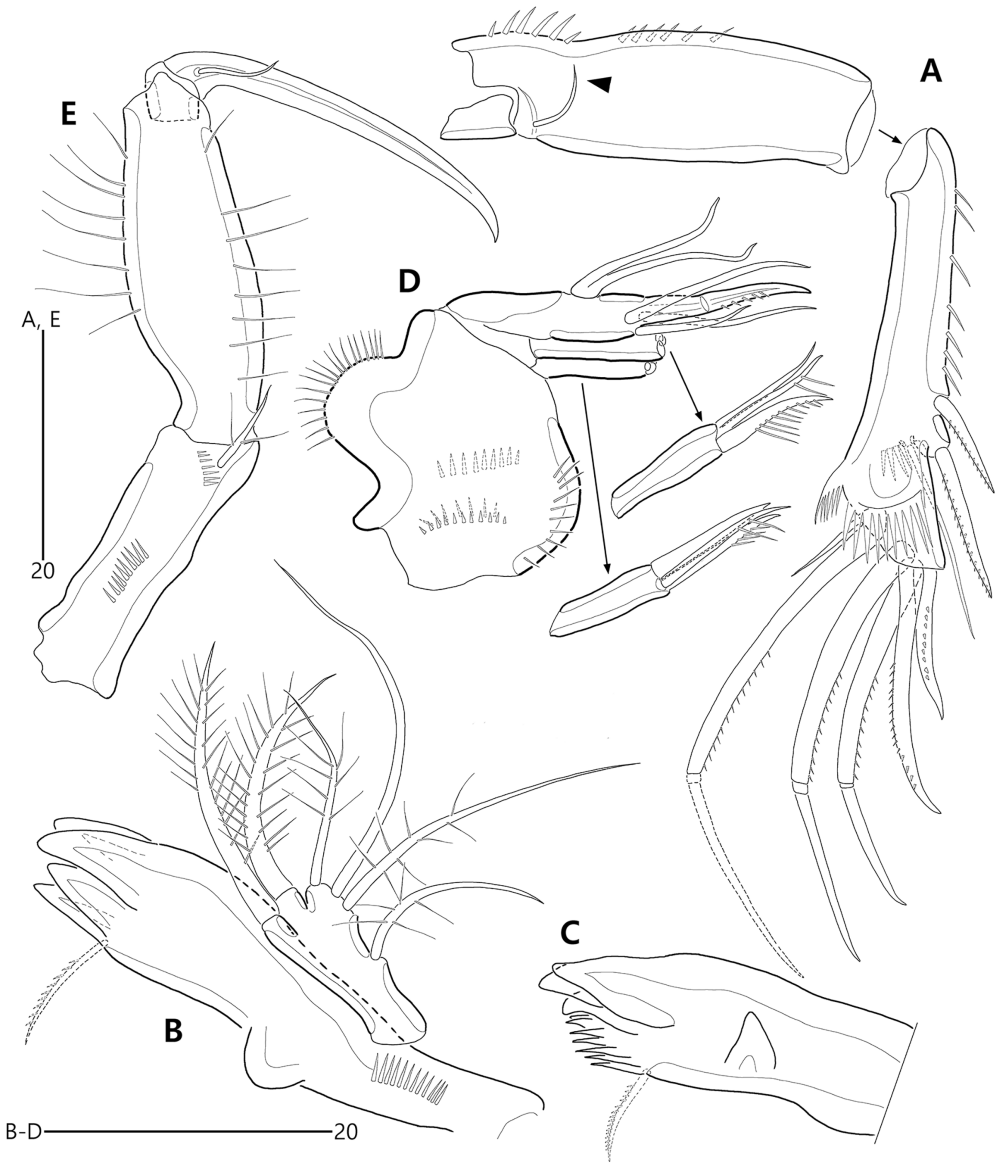
*Bicorniphontodes lacuna* sp. nov., female, holotype (A–E). (A) Antenna; (B) Mandible; (C) Mandibular gnathobase; (D) Maxilla; (E) Maxilliped. Arrowhead indicates the exopod of the antenna.

**Figure 6:**
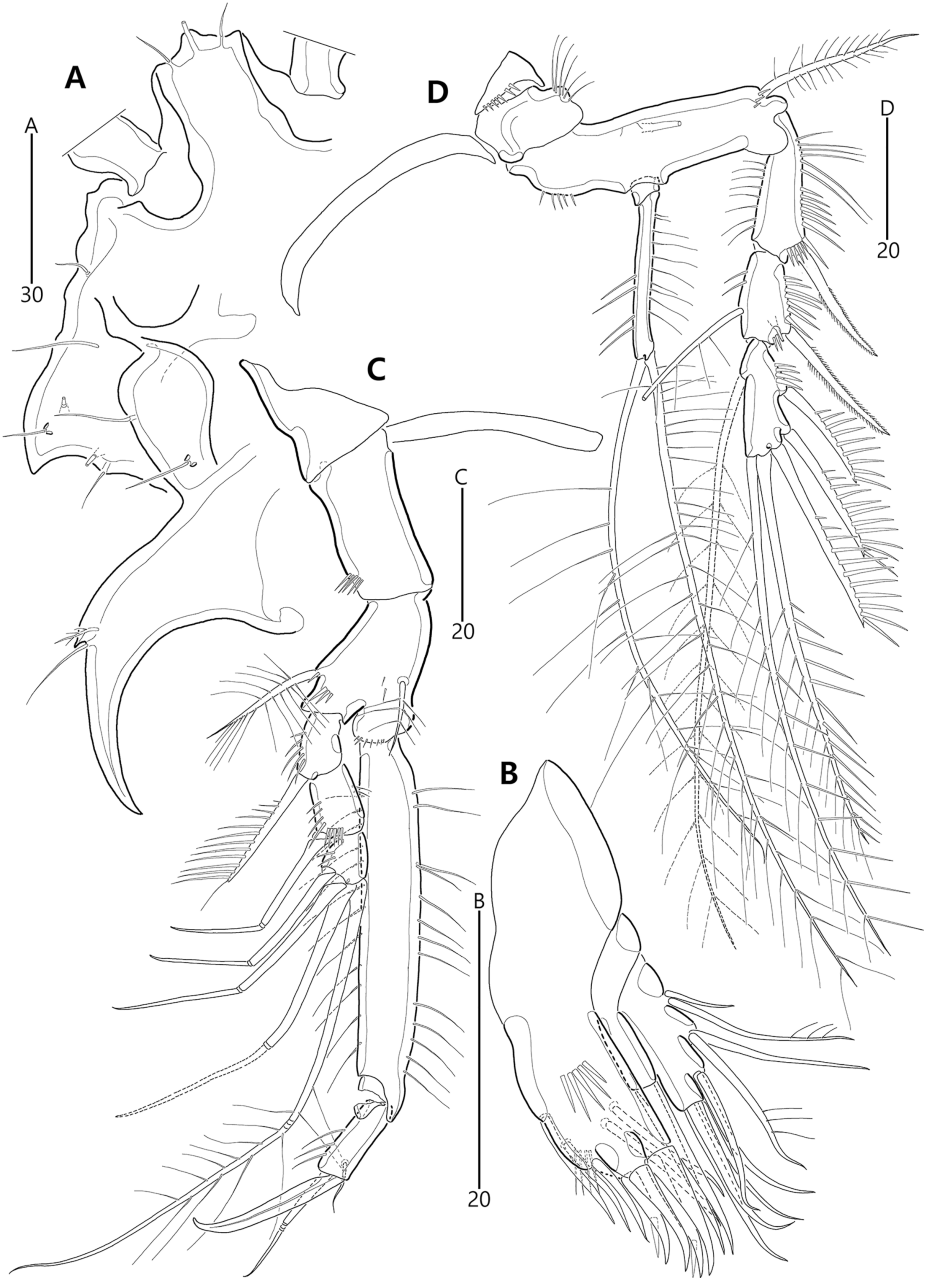
*Bicorniphontodes lacuna* sp. nov., female, holotype (A–D). (A) Rostrum, right mediolateral extension and posterolateral corniform process, ventral; (B) Maxillule; (C) P1, anterior; (D) P2, anterior.

**Figure 7:**
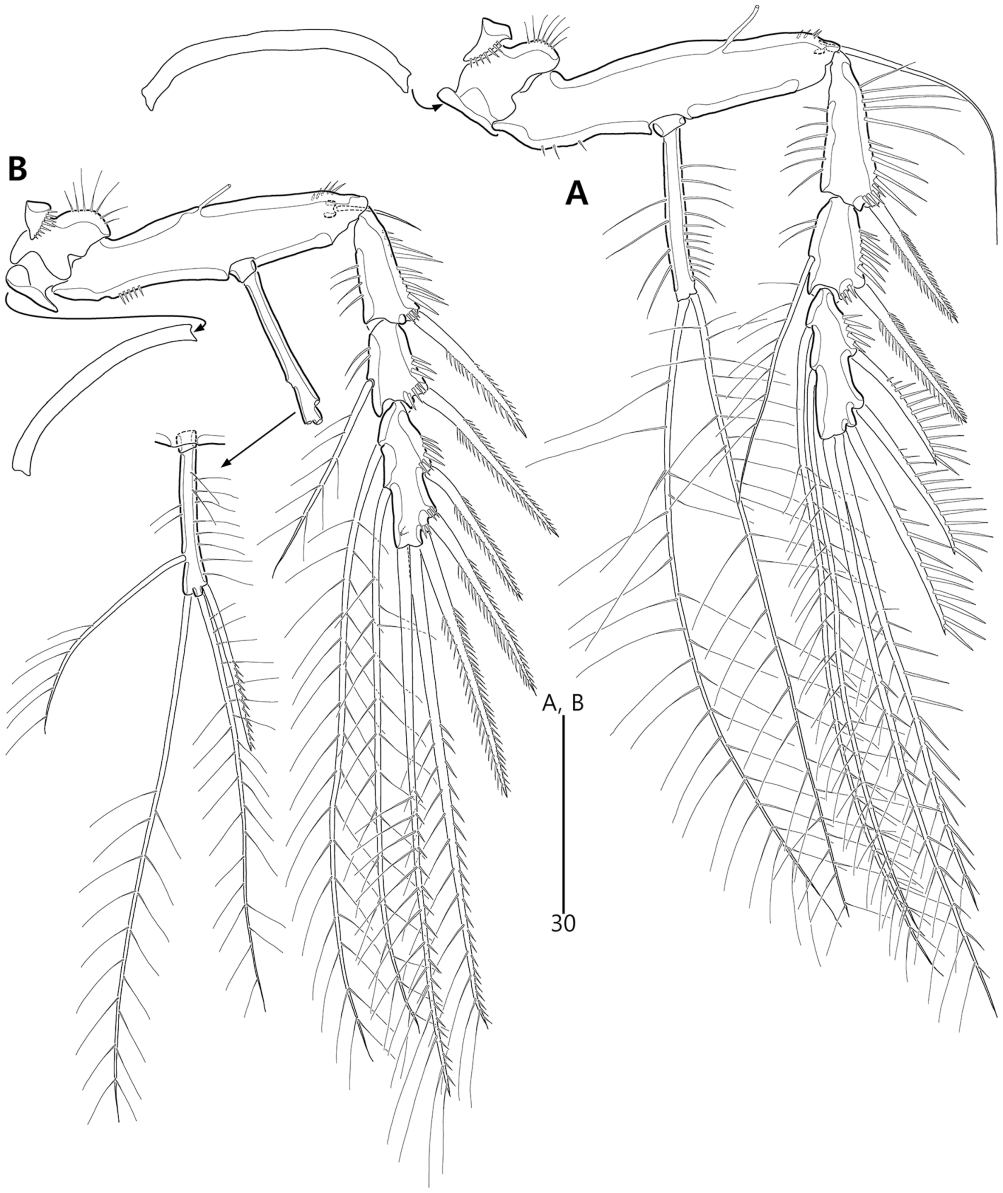
*Bicorniphontodes lacuna* sp. nov., female, holotype (A–B). (A) P3, anterior; (B) P4, anterior.

**Figure 8:**
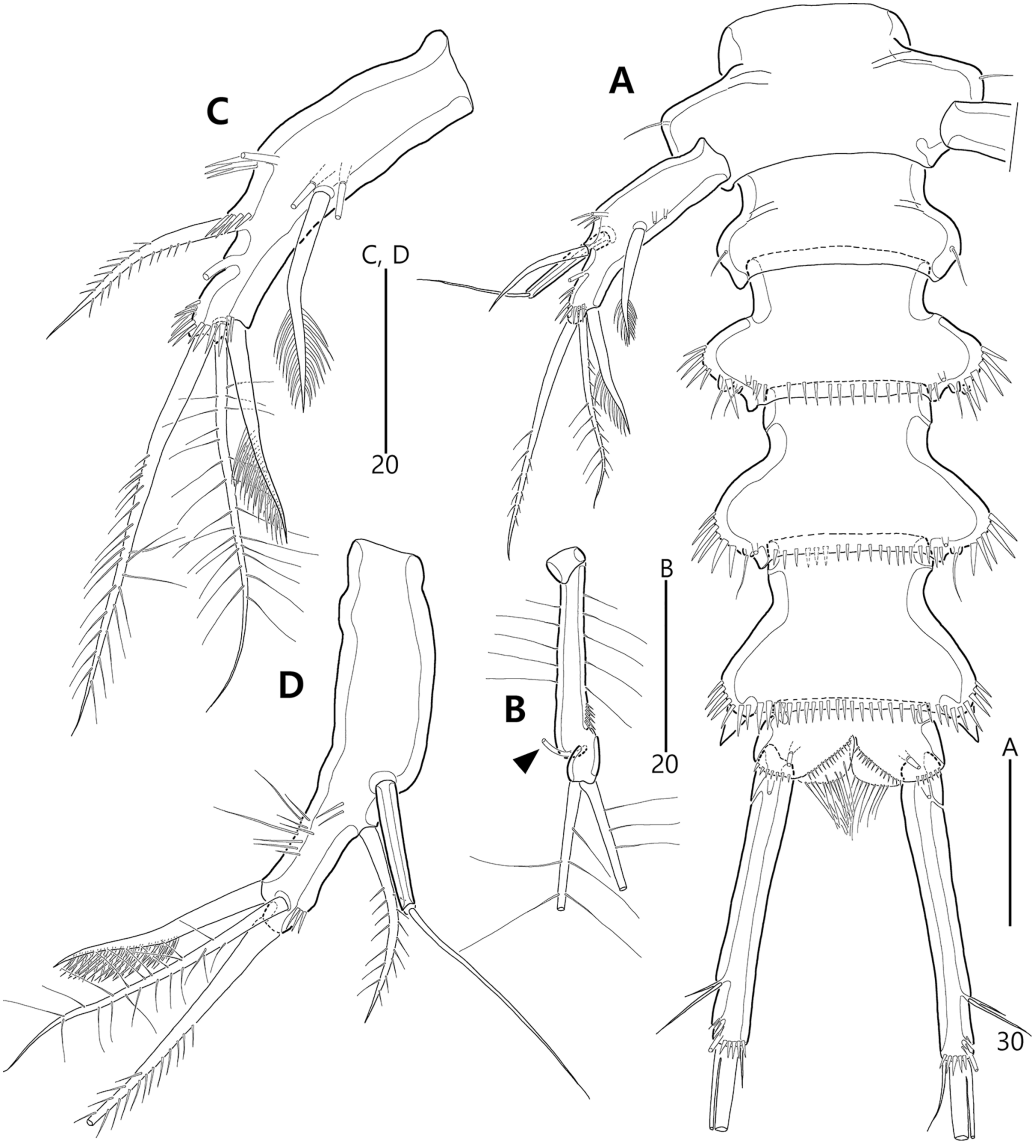
*Bicorniphontodes lacuna* sp. nov., male, paratype 1 (A–D). (A) Urosome, ventral; (B) P3 endopod, anterior; (C) P5, ventral; (D) P5, dorsal. Arrowhead indicates an apomorphy of *B*. *lacuna* sp. nov.

Type material.—Holotype: ♀ dissected on 12 slides (MABIK CR00248414), the subtidal zone off Gajae Rock of Dokdo Island (Liancourt Rocks), Ulleung-eup, Ulleung-gun, Gyeongsangbuk-do, South Korea, 37°14′48.5″N, 131°51′50.4″E, 20 m depth, shell gravel, sampled by SCUBA diving, H.S. Rho leg., August 24, 2016. Paratypes: 1♂ dissected on 12 slides (paratype 1, MABIK CR00248415), 2♀♀, 1♂ dissected on each slide (paratypes 2–4, MInRB-Hr72-S003–MInRB-Hr72-S005), 5♀♀, 1♂ preserved together in a vial with 99% ethanol (Paratypes 5–10, MInRB-Hr72-L001), collection data as holotype.

Additional material examined.—2♀♀, 1♂ dissected on each slide (MInRB-Hr72-S006– MInRB-Hr72-S008), 10♀♀ preserved in a vial with 99% ethanol (MInRB-Hr72-L002), Seobinbaeksa Beach of Udo Island, Udo-myeon, Jeju-si, Jeju-do, South Korea, 33°30′07.2″N, 126°56′34.3″E, 0–1 m depth, coral sand, sampled by a hand net with 50 µm mesh, J.G. Kim leg., June 24, 2014; 1♀, 1♂ preserved in a vial with 99% ethanol (MInRB-Hr72-L003), the sublittoral zone off Beomseom Island, Seogwipo-si, Jeju-do, South Korea, 33°13′10.0″N, 126°31′03.0″E, 20 m depth, sandy sediments including shell gravel, sampled by SCUBA diving, J.G. Kim leg., July 26, 2018.

Description of female (based on the holotype MABIK CR00248414).—Total length 437 µm from tip of rostrum to posterior end of caudal rami in dorsal view (range = 339–437 µm; mean = 396 µm, *n* = 8). Body (Fig. 2A) cylindrical, tapering posteriorly.

Rostrum (Figs. 2A, 6A) distinctly prominent, basally fused to cephalic shield; apical tip weakly produced, with two sensilla and one middorsal tube pore; lateral margins with one pair of pores.

Prosome (Fig. 2A) composed of cephalothorax and three free pedigerous somites. Cephalothorax (Figs. 2A, 6A) wide, about 28% of body length, with one pair of mediolateral triangular extensions and one pair of backwardly curved posterolateral corniform processes; mediolateral extensions with pointed distal tip and two small protuberances (indicated by arrowheads in Fig. 2A) posteriorly; posterolateral corniform processes well-developed, with one small notch bearing one sensillum in the middle of the outer margin and one distinct sensillum-bearing socle on inner margin; dorsal surface covered with several sensilla and pores, and with anterior and posterior cuticular ridges; anterior cuticular ridges distinctly concave; posterior transverse dorsal ridges with four sensillum-bearing socles. Pedigerous somites bearing P2–P4, narrower than cephalothorax, laterally protruded; dorsal surface with one middorsal pore and paired short cuticular ridges on anterior half, and broadly sclerotized dorsal area on posterior half; dorsal sclerotized area subdistally with two sensilla and distally with six, four, and four sensillum-bearing socles, respectively; laterally with one pore, one sensillum and one sensillum-bearing socle; hyaline frills serrate.

Urosome (Figs. 2A, 3A) composed of P5-bearing somite, genital double-somite and three abdominal somites. P5-bearing somite more protruded laterally than preceding somite, with one middorsal pore, one pair of dorsolateral pores, one pair of lateral sensilla and four dorsal sensillum-bearing socles; hyaline frill as those of prosomites. Genital double-somite completely fused, distinctly contracted as furrows probably indicating original division (see arrowheads in Fig. 2A, 3A); genital half with one middorsal pore, one pair of dorsolateral sensillum-bearing socles, one pair of lateral sensilla, and one pair of posterolateral protuberances carrying sensillum; first abdominal somite dorsally with one mid pore, dorsolaterally with one pair of tube pores, and two pairs of sensillum-bearing socles, and laterally with one pair of spinular rows and one pair of sensillum-bearing socles on posterior half; dorsal surfaces of genital and first abdominal somites with short cuticular ridges; hyaline frill ornamented with distinct protuberances bearing two or three lappets. Genital apertures (Figs. 3A, 3B) fused medially, forming transverse genital slit and covered by single plate (vestigial P6) carrying one lateral seta on both sides; copulatory pore large, posterior to genital slit. Second abdominal somite posteriorly expanded, ornamented as in preceding one, dorsally with longitudinal cuticular ridges. Penultimate somite posteriorly expanded, distinctly protruded at posterolateral corners; dorsally with longitudinal cuticular ridges and one pair of pores and ventrally with one row of spinules; hyaline frill serrate. Anal somite (Figs. 2A, 3A) small, with one pair of dorsolateral pores, one pair of ventrolateral pores, and one pair of ventral tube pores; ventral surface ornamented with one pair of spinular rows near pedestal of insertion of caudal rami and one pair of oblique spinular rows near medial cleft; anal opening armed with fine long setules; anal operculum (Fig. 3D) distinctly prominent, with serrate posterior margin, and accompanied by one pair of sensilla.

Caudal rami (Figs. 2A, 3A, 3C) slightly divergent, elongate, about eight times as long as wide, ornamented proximally with one lateral and one ventral tube pore, and subdistally with one lateral pore, one ventral tube pore and one row of stout ventral spinules; with seven setae: setae I and II inserted at distal fifth of lateral margin, dorsolateral seta II twice as long as ventrolateral seta I; seta III arising from dorsolateral surface subdistally, about 1.5 times as long as seta II; terminal setae IV and V fused basally; seta IV unipinnate, as long as caudal ramus; seta V bipinnate, about three times as long as seta IV; seta VI inserted at distal inner corner, slightly longer than seta I; tri-articulate seta VII arising from a small peduncle on dorsal surface subdistally, as long as caudal rami.

Antennule (Fig. 4A) five-segmented. First segment with one weak outer protuberance surrounded by spinules, two rows of inner spinules, and one plumose inner seta. Second segment larger than preceding one; inner margin with several spinules, outer margin with a distinct bump bearing long spinules, and anterior surface with one tube pore and one small protuberance (near two peduncles bearing long setae); with two plumose setae and seven bare setae. Third segment longest, with five bare inner setae; distal inner corner posteriorly with one long bare seta and one peduncle carrying one aesthetasc and one long bare seta. Fourth segment smallest, with one long bare inner seta. Distal segment with three bare inner setae, one bi-articulate outer seta, one bare and two bi-articulate anterior setae subapically, two bi-articulate apical setae; with one apical acrothek composed of one aesthetasc and two long setae. Setal formula: 1-[1], 2-[9], 3-[6 + (1 + aesthetasc)], 4-[1], 5-[9 + acrothek].

Antenna (Fig. 5A) three-segmented. Coxa small, unornamented (damaged during dissection process). Allobasis elongate, ornamented with two rows of spinules along abexopodal margin. Exopod represented by one small seta (see arrowhead in Fig. 5A). Endopod as long as allobasis, ornamented with inner spinules, one group of anterior spinules, and two spinulose subdistal frills; lateral armature composed of two unipinnate spines and one delicate seta, and distal armature comprising two unipinnate spines, three geniculate setae, and one tiny seta which is fused basally to adjoining longest geniculate seta.

Mandible (Figs. 5B, 5C). Coxa with one bulge medially and one row of spinules proximally; slender gnathobase armed with multicuspidate teeth and one unipinnate seta (lost during a dissecting process in holotype; present in a few paratypes). Palp one-segmented, with two basal plumose setae; exopod represented by one plumose seta and endopod represented by one bare and two plumose setae.

Maxillule (Fig. 6B). Praecoxal arthrite ornamented with one row of posterior spinules and one row of anterior spinules subdistally; with four distal spines (of which anterior two ones bearing one large spinule subdistally), three lateral setae, and two juxtaposed anterior setae. Coxal endite with two bare distal setae. Basis with two endites; distal and lateral ones with three and two bare setae, respectively. Both rami incorporated into basis; exopod represented by one small and one long uniplumose seta; endopod represented by one long bare and one long uniplumose seta.

Maxilla (Fig. 5D). Syncoxa ornamented with spinules along outer and inner margins, one row of anterior spinules, and two rows of posterior spinules; with two endites: proximal endite with two unipinnate setae (of which proximal one fused to endite basally) and one bare seta; distal endite with one bare and two unipinnate setae. Allobasis drawn out into stout unipinnate claw, with three accompanying bare setae. Endopod represented by two bare setae (fused basally).

Maxilliped (Fig. 5E) three-segmented. Syncoxa elongate, with two rows of spinules and one weakly plumose subdistal seta on medial surface. Basis longer than syncoxa; inner and outer margins slightly convex, ornamented with long setules. Endopod one-segmented, small, with one stout claw as long as basis, accompanied by one small seta.

P1 (Fig. 6C). Praecoxa large, triangular, unornamented. Intercoxal sclerite transversely elongate. Coxa elongate, with one row of outer spinules subdistally. Basis elongate, with one plumose outer seta accompanied by few spinules at its base, and few anterior spinules; inner seta plumose, displaced onto anterior surface near inner margin; pedestal for insertion of inner ramus well-developed, with spinules distally. Exopod three-segmented, reaching 2/5 of enp-1; exp-1 with outer spinules and one unipinnate and comb-like outer spine; exp-2 with inner and outer spinules, and one geniculate outer seta; exp-3 smallest, with few outer spinules and four (two outer and two distal) geniculate setae (of which innermost one longest and plumose at distal half). Endopod prehensile, two-segmented; enp-1 elongate, about twice as long as exopod, with outer and inner setules; enp-2 small, with outer setules, one stout spine and one geniculate seta distally, and one tiny posterior seta subapically.

P2–P4 (Figs. 6D, 7A, 7B). Praecoxae small, with spinules distally. Intercoxal sclerite transversely elongate. Coxae with one row of outer setules. Bases transversely elongate, with few inner spinules proximally; with one well-developed tube pore on anterior surface (in P2) or outer margin (in P3–P4), and outer seta (plumose in P2 and bare in P3–P4) accompanied by set of spinules basally. Exopods three-segmented; exp-1 with two rows of outer spinules and one row of inner setules, and with one pinnate outer spine accompanied by set of spinules basally; exp-2 with one row of outer spinules and few inner setules, and with one pinnate outer spine accompanied by set of spinules basally and one uni- or bi-plumose inner seta; exp-3 with one row of outer spinules proximally, with three outer spines (strongly unipinnate and comb-like in P2–P3 and bi-pinnate in P4), two distal setae (ornamented with outer spinules and inner setules), and one (in P2) or two (in P3–P4) plumose inner setae; one tube pore present on P2 exp-2 and one pore present on P4 exp-3 Endopods two-segmented; enp-1 small, unornamented; enp-2 elongate, with outer and inner setules; P2–P3 enp-2 with two plumose setae distally, P4 enp-2 with one unipinnate outer spine, two plumose distal setae, and one uniplumose inner seta.

P5 (Figs. 3A, 3E, 3F) two-segmented. Baseoendopod elongate, with two groups of setules and one subapical tube pore on anterior margin, one row of dorsal spinules near posterior margin, and two ventral tube pores; with elongate setophore carrying one bare seta. Endopodal lobe completely incorporated into baseoendopod, represented by two fishbone-like spines. Exopod shorter than baseoendopod, about 0.7 times as long as baseoendopod, with one row of long anterior setules and one ventral tube pore; with one plumose anterior and one posterior seta proximally, one pinnate dorsal seta subapically, and one long plumose and one pinnate apical seta.

Description of male (based on paratype 1 MABIK CR00248415). Total body length smaller than female, about 386 µm (range = 331–386 µm; mean = 359 µm, *n* = 2) (Fig. 2B). Sexual dimorphic differences detected in cephalothorax, urosome, antennule, P3 endopod, P5 and P6.

Cephalothorax (Fig. 2B). Anterior one of two protuberances on mediolateral triangular extensions larger than that of female (see arrowheads in Fig. 2B) and posterolateral corniform processes less curved and smaller than in the female.

Urosome (Figs. 2B, 8A) six-segmented, genital and first abdominal somites separated.

Antennule (Fig. 4B) subchirocer, seven-segmented. First segment with one weak anterior protuberance ornamented with spinules and one row of outer spinules, and two rows of inner spinules; with one plumose inner seta basally accompanied by one row of posterior spinules. Second segment with one outer bump ornamented with setules, one plumose and six bare inner setae, two anterior setae, and one small anterior protuberance. Third segment with six bare inner setae subdistally and distally. Fourth segment represented by small sclerite, with two bare setae. Fifth segment swollen; posterior surface proximally with one small spinulose spine, one uniplumose and five bare setae, medially with one uniplumose and two bare setae, and subdistally with two long bare setae and one peduncle carrying one aesthetasc and one long seta (fused basally); inner margin with serrate protrusions proximally and small denticles anteriorly and posteriorly. Sixth segment small, with three inner setae. Seventh segment conical in shape, with one bare inner seta, three bi-articulate anterior setae, and two bi-articulate outer setae; anterior surface with one acrothek comprising one aesthetasc and two bare setae. Setal formula: 1-[1], 2-[9], 3-[6], 4-[2], 5-[12 + (1 + aesthetasc)], 6-[3], 7-[6 + acrothek].

P3 endopod (Fig. 8B) two-segmented, but the incomplete division of original second and third segments present; enp-1 small, unornamented; original enp-2 elongate, ornamented with outer and inner setules and outer spinules, and apically with one tube pore (see arrowhead in Fig. 8B); original enp-3 small, probably fused to enp-2 basally, apically with two plumose setae.

P5 (Figs. 8A, 8C, 8D). Exopod fused to baseoendopod, forming single lobe; anterior margin with three groups of spinules and two tube pores, and one pinnate seta; posterior margin subapically with one fishbone-like seta; distal margin with one plumose seta; dorsal surface with few spinules, medially with elongate setophore carrying one bare seta, and subdistally with one plumose seta; ventral surface medially with two tube pores and one fishbone-like seta, and subapically with one row of spinules.

P6 (Fig. 8A) completely fused to somite, without any vestiges.

Variability. The morphological variation of *B*. *lacuna* sp. nov. was observed in the shape of the anal operculum. The spinular arrangement (the number of elements and their size) on the distal margin is variable (see Figs. 3D, 3G, 3H).

Etymology. The specific name “lacuna” is derived from the Latin noun *lacuna*, meaning “cavity” or “hollow”. It alludes to the typical shape of the female genital double-somite, which has distinct lateral furrows. It is in the nominative singular and is to be treated as a noun in apposition.

Remarks. *Bicorniphontodes lacuna* sp. nov. is reminiscent of *B*. *clarae* and *B*. *comptus* sp. nov. in terms of having a cephalothorax with dorsal cuticular ridges and distinctly curved backwardly posterolateral corniform processes. However, this new species differs from *B*. *clarae* in the following: the rostrum has a blunt distal margin with a central ventral tube pore (*vs*. prominent and elongated, and without the tube pore in *B*. *clarae*); the distal margin of the antennary endopod has a delicate seta accompanied by the subapical longest geniculate spine (*vs*. absent in *B*. *clarae*); the maxillular exopod and endopod are both represented by two setae (*vs*. a single seta in *B*. *clarae*); the maxillipedal syncoxa has a subdistal seta (*vs*. absent in *B*. *clarae*); and the anal operculum is armed with triangular processes (*vs*. fine setules in *B*. *clarae*). With the exception of the anal operculum, these morphological features are displayed in another Korean congener, *B*. *comptus* sp. nov.; thus, *B*. *lacuna* sp. nov. probably appears to be most similar to *B*. *comptus* sp. nov. However, the female genital double-somite shape in *B*. *lacuna* sp. nov. is more distinctly contracted (nearly forming a furrow; see arrowheads in Figs. 2A, 3A) in the lateral margins, and the caudal rami are much longer than in *B*. *comptus* sp. nov.

Generally, members of *Bicorniphontodes*, as well as the related genera *Laophontodes* and *Paralaophontodes*, exhibit sexual dimorphism in the male P3 endopod. Although females have a two-segmented endopod, the ramus of the male is three-segmented and exhibits an apophysis arising from the distal margin of the second segment. In *B*. *lacuna* sp. nov., however, the division between the distal two segments is probably incomplete, and a tube pore is present where apophysis exists in other congeners (see arrowhead in Fig. 8B). This unique characteristic is considered as a clear autapomorphy of *B*. *lacuna* sp. nov.

*Bicorniphontodes comptus* sp. nov.

urn:lsid:zoobank.org:act:A35DF79E-6E51-49EB-9200-996ECE8F09F9

Figures 9–14

**Figure 9:**
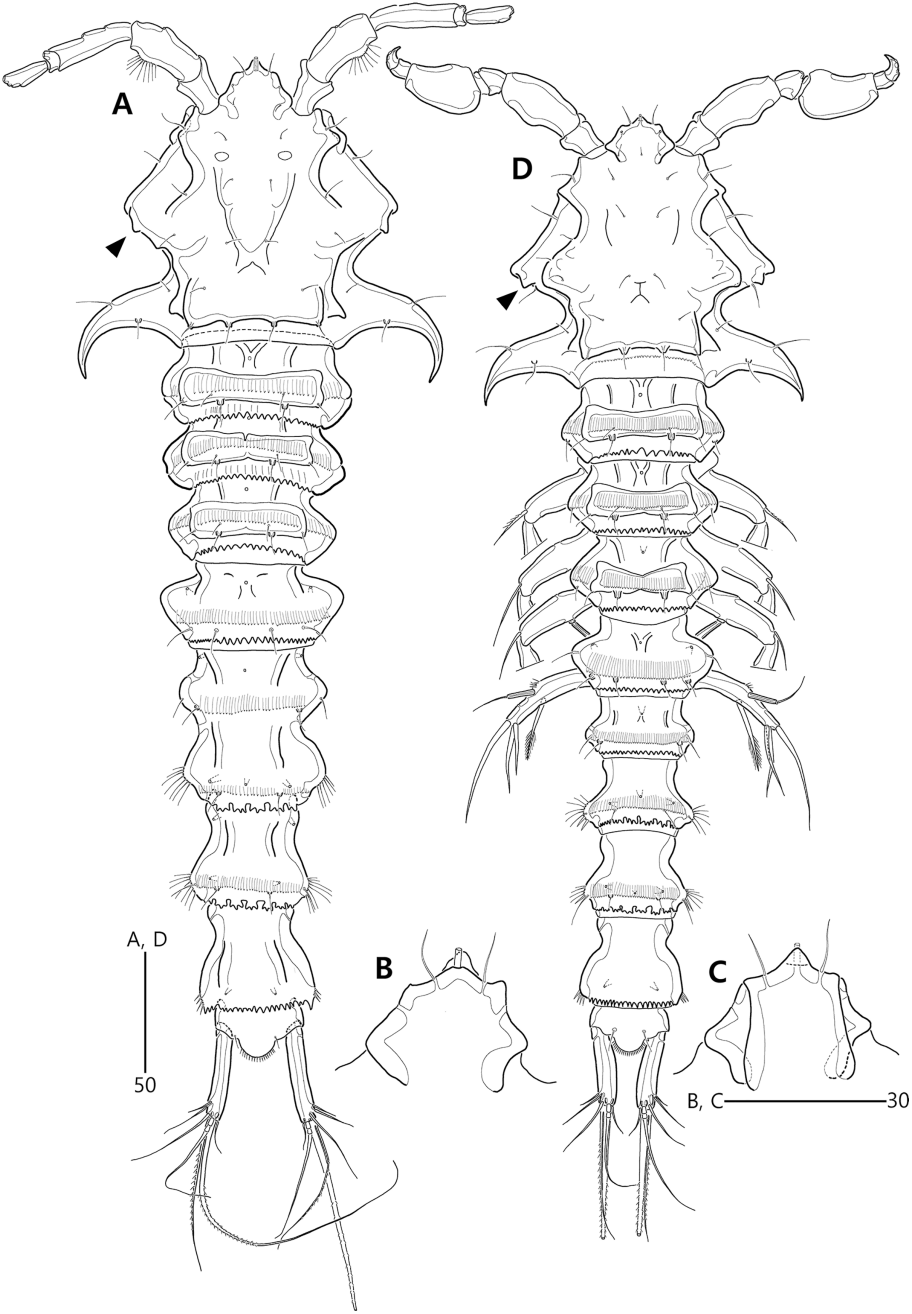
*Bicorniphontodes comptus* sp. nov., female, holotype (A–C), male, paratype 1 (D). (A) Female habitus, dorsal; (B) Rostrum, dorsal; (C) Rostrum, ventral; (D) Male habitus, dorsal. Arrowheads indicate apomorphies of *B*. *comptus* sp. nov.

**Figure 10:**
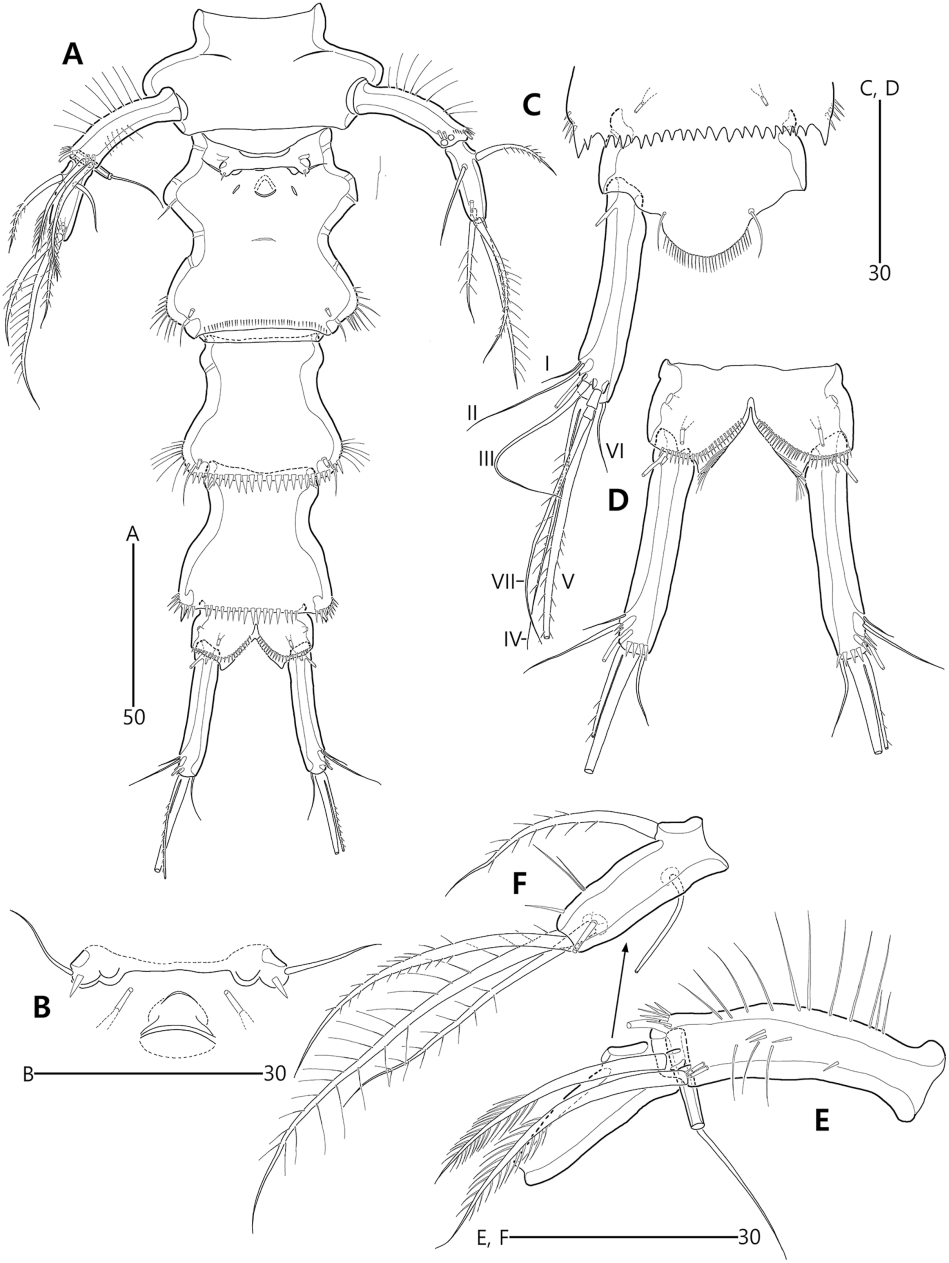
*Bicorniphontodes comptus* sp. nov., female, holotype (A–F). (A) Urosome, ventral; (B) Genital field; (C) Anal somite and right caudal ramus, dorsal; (D) Anal somite and caudal rami, ventral; (E) P5 baseoendopod and exopod omitted its setal armature, ventral; (F) P5 exopod, ventral.

**Figure 11:**
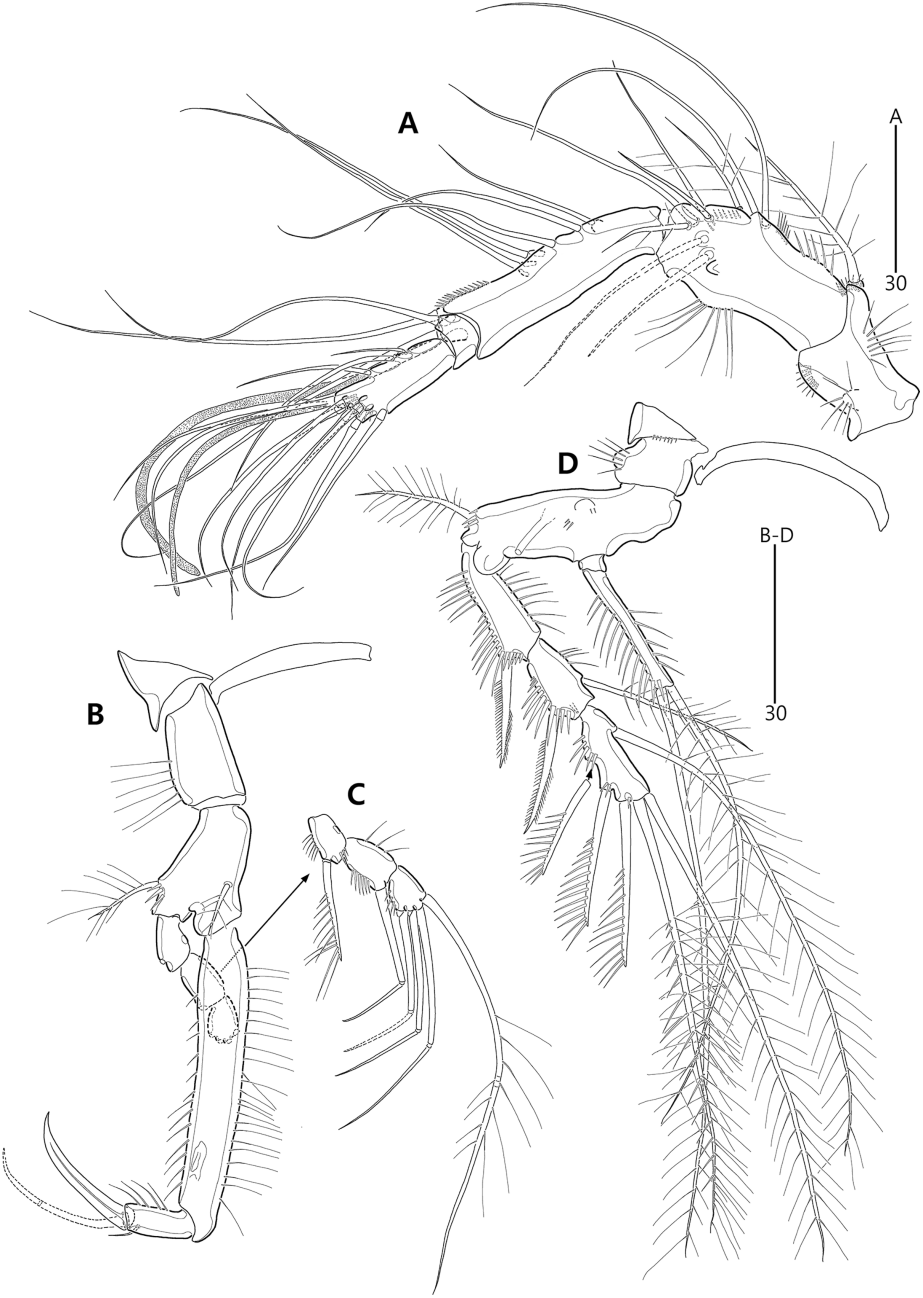
*Bicorniphontodes comptus* sp. nov., female, holotype (A–D). (A) Antennule; (B) P1, anterior; (C) P1 exopod, anterior; (D) P2, anterior.

**Figure 12:**
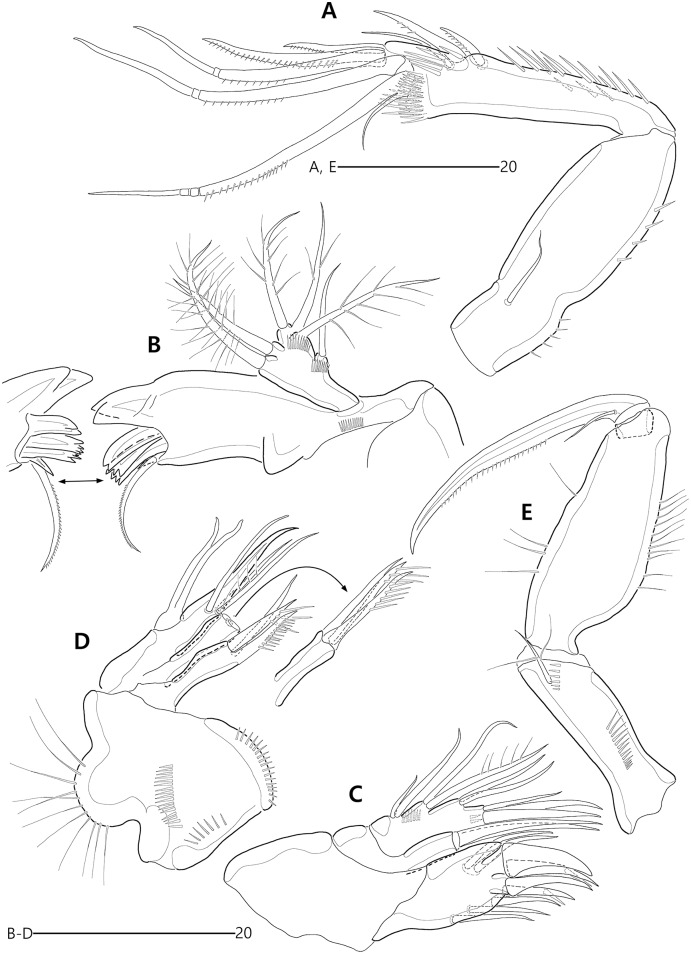
*Bicorniphontodes comptus* sp. nov., female, holotype (A–E). (A) Antenna; (B) Mandible; (C) Maxillule; (D) Maxilla; (E) Maxilliped.

**Figure 13:**
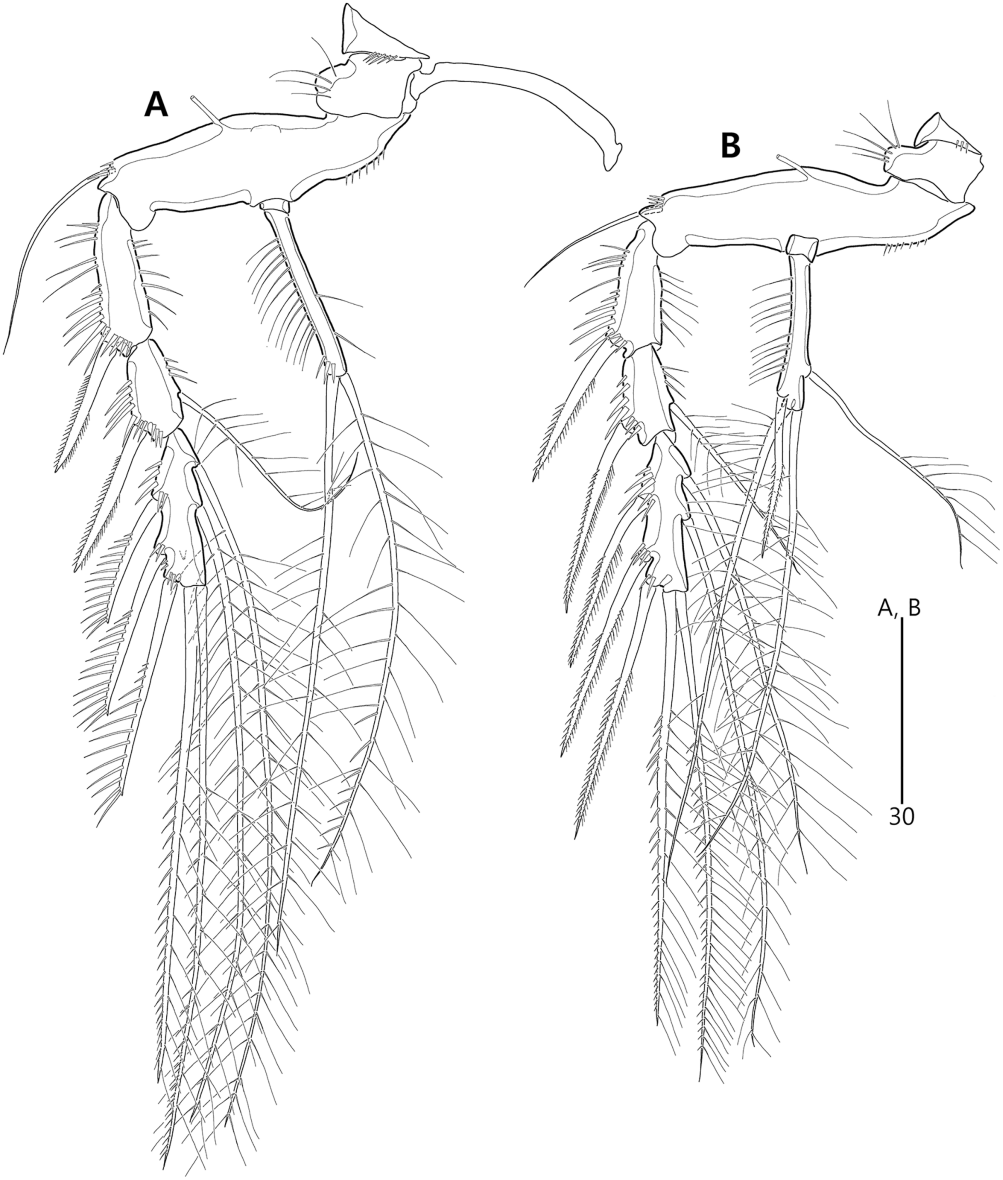
*Bicorniphontodes comptus* sp. nov., female, holotype (A–B). (A) P3, anterior; (B) P4, anterior.

**Figure 14:**
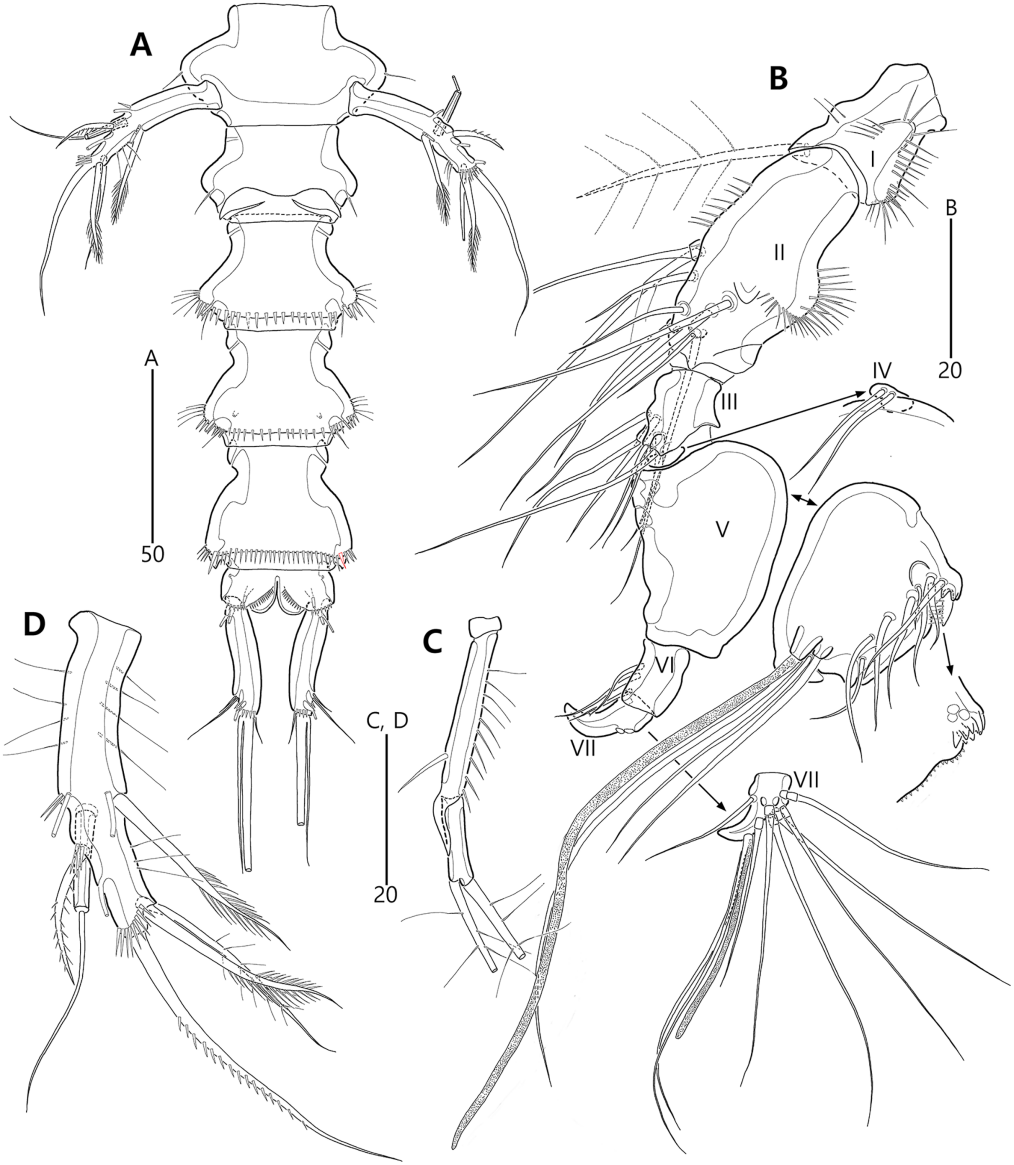
*Bicorniphontodes comptus* sp. nov., male, paratype 1 (A–D). (A) Urosome, ventral; (B) Antennule; (C) P3 endopod; (D) P5, ventral.

Type material.—Holotype: ♀ dissected on 12 slides (MABIK CR00248416), the subtidal zone off Sibri Rock where is located 500 m away from Gyeongpo beach, Gangneung-si, Gangwon-do, South Korea, 37°48′39.9″N, 128°54′42.1″E, 9.6 m depth, sandy sediments, sampled by a Smith-McIntyre grab; J. Lee, H.G. Lee leg., July 25, 2017. Paratypes: 1♂ dissected on 12 slides (paratype 1, MABIK CR00248417), 2♀♀ dissected on each slide (paratypes 2–3, MInRB-Hr73-S003–MInRB-Hr73-S004), 1♀, 1♂ preserved in a vial with 80% ethanol (paratypes 4–5, MInRB-Hr73-L001), collection data as holotype.

Description of female (based on holotype MABIK CR00248416).—Habitus (Fig. 9A) cylindrical, tapering posteriorly; total body length about 446 µm from tip of rostrum to posterior end of caudal rami in dorsal aspect (range = 377–446 µm; mean = 427 µm, *n* = 4); greatest width about 153 µm at posterolateral corniform processes on cephalothorax.

Rostrum (Figs. 9A–9C) distinctly prominent, triangular, basally fused to cephalic shield; apical tip distinctly produced; subapically with two sensilla and one middorsal tube pore, laterally with one pair of pores.

Cephalothorax (Fig. 9A) extended medially, with one pair of mediolateral triangular extensions and one pair of backwardly curved posterolateral corniform processes; dorsal surface with lateral longitudinal and median U-shaped cuticular ridges, and posterior protrusion bearing four sensillum-bearing socles; mediolateral extensions with blunt tip and small posterior protrusion (see arrowhead in Fig. 9A); posterolateral corniform processes well-developed as preceding species. Free pedigerous somites (Fig. 9A) extended laterally; dorsal surfaces anteriorly with one median pore and two (in P3–P4-bearing somites) or three (in P2-bearing somite) ridges, posteriorly with sclerotized area with two sensilla subdistally, and four sensillum-bearing socles (of which lateral ones are weakly developed) distally; hyaline frills serrate as preceding species.

P5-bearing somite (Figs. 9A, 10A) inverse trapezoidal in dorsal aspect, more extended laterally than free pedigerous somites; dorsal surface anteriorly with one median pore and one pair of dorsolateral pores, and posteriorly with four sensilla; hyaline frill as in prosomites. Genital and first abdominal somites completely fused, forming genital-double somite (Figs. 9A, 10A), but smoothly contracted laterally and original division probably indicated by transverse row of minute spinules; original genital somite slightly swollen laterally, anteriorly with dorsal ridges and one middorsal pore, laterally with two pairs of pores and one pair of sensilla, and dorsolaterally with one pair of sensillum-bearing socles; original first abdominal somite swollen laterally, anteriorly with dorsal ridges, laterally with one pair of setular rows posteriorly, and dorsally with three pores and two sensillum-bearing socles; hyaline frill composed of five distinct projections with two or three lappets; ventral surface posteriorly with one pair of tube pores and one row of fine spinules. Genital field (Figs. 10A, 10B) located at anterior half of genital somite; both gonopores fused medially, forming genital slit covered by single operculum derived from vestigial P6, with one seta and one spinule on both sides; copulatory pore large, located in median depression, accompanied by one pair of tube pores laterally. Second abdominal somite posterolaterally expanded; dorsal surface anteriorly with two cuticular ridges and two lateral pores, posteriorly with two medial sensillum-bearing socles and two pores; lateral margins posteriorly with one pair of setular rows and one pair of sensilla; hyaline frill ornamented with seven projections with two or three lappets; ventral surface posteriorly with one pair of tube pores and one row of stout spinules. Penultimate somite as preceding one in shape; dorsal surface with two cuticular ridges medially and two pores posteriorly; lateral margins with one pair of pores anteriorly and one pair of spinular rows posteriorly; both sides of posterior margin distinctly produced, with one row of ventral spinules; hyaline frill as those of free pedigerous somites; ventral surface with one row of posterior spinules. Anal somite (Figs. 9A, 10A, 10C, 10D) small, with one pair of lateral pores, one pair of large ventrolateral pores, and one pair of ventral tube pores; ventral surface ornamented with two pairs of spinular rows posteriorly; anal opening ornamented with fine long setules; anal operculum distinctly prominent, ornamented with fine spinules, accompanied by one pair of long sensilla.

Caudal rami (Figs. 9A, 10A, 10C, 10D) elongate, slightly divergent, slightly shorter than penultimate somite, about five times as long as wide; ornamentation and setal position as preceding species; seta II about three times as long as seta I; seta III as long as caudal ramus; seta V about twice as long as seta IV; seta VI about twice as long as seta I; tri-articulate seta VII slightly longer than seta IV.

Antennule (Fig. 11A) five-segmented. First segment with one small anterior protuberance with setules, one row of outer spinules posteriorly, and one row of inner setules; with one plumose inner seta accompanied by spinular row basally. Second segment with three rows of inner spinules and one row of setules on outer bump; inner margin with three plumose and four bare setae; anterior surface with two long bare setae and one small protuberance. Third segment elongate, with one row of inner spinules subdistally; with five inner setae and one long inner bare seta subdistally; distal margin ventrally produced into pedestal, with one aesthetasc and one long seta (fused basally) on its tip. Fourth segment small, with one long bare inner seta. Distal segment about four times as long as preceding segment, with ventral spinules subdistally; with three bare inner setae, one bi-articulate outer seta; distal and subdistal margins with one bare seta and four bi-articulate elements, and acrothek (one aesthetasc and two long bare setae). Setal formula: 1-[1], 2-[9], 3-[6 + (1 + aesthetasc)], 4-[1], 5-[9 + acrothek].

Antenna (Fig. 12A) three-segmented. Coxa lost during dissection process. Basis and original first endopodal segment fused forming allobasis; elongate, ornamented with two rows of abexopodal spinules (one row present on original basis and another present on original first endopodal segment). Exopod represented by one small bare seta. Free endopodal segment as long as but narrower than allobasis, ornamented with inner spinules, one row of anterior spinules subdistally, and two spinulose subdistal frills; inner margin subdistally with one delicate seta and two unipinnate spines; distal margin with two unipinnate spines, three geniculate setae, and one small seta fused basally to adjoining longest geniculate seta.

Mandible (Fig. 12B). Coxa with one bulge medially and ornamented with one row of spinules proximally; gnathobase armed with multicuspidate teeth and one unipinnate seta. Both rami incorporated into basis forming one-segmented palp, with two rows of spinules; original basis with two plumose setae distally; exopod small, with one bare seta apically; endopod larger than exopod, distally with three plumose setae.

Maxillule (Fig. 12C). Praecoxa unornamented; arthrite well-developed, distally with two bare posterior spines and two anterior spines (bearing one spinule), laterally with three bare setae, and anteriorly with two juxtaposed setae. Coxal endite elongate, with two long setae distally. Basis, exopod and endopod fused, forming single lobe. Basis ornamented with few anterior spinules subdistally and proximally; distally with one delicate and two stout setae, and laterally with two bare setae. Exopod represented by one small and one long seta, and endopod represented by two long (one bare and one uniplumose) setae.

Maxilla (Fig. 12D). Syncoxa ornamented with one row of long setules along bump on outer margin, one row of spinules on inner margin, and two rows of spinules on surface; with two endites: proximal endite distally with one bare seta, two unipinnate setae (of which proximal one fused to endite basally); distal endite with one bare and two unipinnate setae. Allobasis drawn out into stout claw accompanied by one uniplumose and two bare setae. Endopod represented by two long bare setae (fused basally).

Maxilliped (Fig. 12E). Syncoxa elongate, ornamented with one row of medial spinules; subdistally with one weakly plumose seta, accompanied by one group of medial spinules at its base. Basis longer than syncoxa; outer margin convex medially, ornamented with setules; palmar margin nearby straight, weakly ornamented with setules. Endopod small, one-segmented, with one stout unipinnate claw as long as basis, accompanied by one tiny seta.

P1 (Figs. 11B, 11C). Praecoxa triangular, unornamented. Intercoxal sclerite transversely elongate. Coxa elongate, about twice as long as wide, with long outer setules. Basis longer and wider than preceding segment; outer margin with one plumose seta carrying one row of anterior spinules at its base; anterior surface with one plumose seta near inner margin; pedestal for insertion of inner ramus well-developed, unornamented. Exopod three-segmented, reaching to proximal third of enp-1; exp-1 with one row of outer spinules, one row of distal spinules, and one unipinnate and comb-like outer spine; exp-2 longest, ornamented with outer spinules and inner setules, with one geniculate outer seta; exp-3 smallest, with one row of outer spinules, one long plumose and three bare geniculate setae. Endopod prehensile, two-segmented; enp-1 elongate, slightly over twice as long as exopod, ornamented with outer spinules and inner setules; enp-2 distinctly smaller than preceding segment, with one row of outer setules, one tiny inner seta posteriorly and subdistally, and one stout spine and one geniculate seta (broken during a dissecting process) distally.

P2–P4 (Figs. 11D, 13A, 13B). Praecoxae small, distally with spinules. Intercoxal sclerite transversely elongate. Coxae with one row of outer setules. Bases transversely elongate; outer seta plumose in P2 and bare in P3–P4, bearing set of spinules at its base; anterior surface of P2 ornamented with few spinules and one strong tube pore; outer margins of P3–P4 with one tube pore; inner margins of P3–P4 ornamented with fine spinules proximally. Exopods three-segmented; exp-1 elongate, ornamented with two rows of outer spinules and one row of inner setules, with one pinnate outer spine carrying few spinules at its base; exp-2 smallest, ornamented with one row of outer spinules and one row of inner spinules, with one pinnate outer spine carrying few spinules at its base, and one plumose inner seta; P2 exp-2 with, P3–P4 exp-2 without anterior pore; exp-3 elongate, ornamented with one row of outer spinules proximally, with three outer spines (strongly unipinnate and comb-like in P2–P3 and bipinnate in P4) carrying set of few spinules at each base, two distal setae (ornamented with outer spinules and inner setules), and one (in P2) or two (in P3–P4) plumose inner setae; P3 exp-3 with, P2 and P4 exp-3 without anterior pore. Endopods two-segmented, almost reaching to distal end of exp-2; enp-1 small, unornamented; enp-2 elongate, outer margin with one row of setules and one group of subdistal spinules in P2–P3, and inner margin with one row of spinules in P2, few spinules in P3 and unornamented in P4; P2–P3 enp-2 with two plumose setae distally; P4 enp-2 with one pinnate outer spine, two plumose distal setae and one uniplumose inner seta.

P5 (Figs. 10A, 10E, 10F) two-segmented. Baseoendopod elongate, anterior margin medially with one row of setules, and subdistally with one tube pore and one set of spinules; ventral surface with few spinules and setules, and with two subdistal tube pores; outer setophore elongate, with one long bare seta. Endopodal lobe completely incorporated into baseoendopod, represented by two fishbone-like spines. Exopod about 0.6 times as long as baseoendopod, anterior margin with two groups of spinules subdistally and one pinnate seta proximally; distal margin with one pinnate and one long plumose seta; dorsal surface with one bare seta proximally and one plumose seta subdistally; ventral surface with one tube pore subdistally.

Description of male (based on paratype 1 MABIK CR00248417). Total body length smaller than female, about 416 µm (range = 377–416 µm; mean = 397 µm, *n* = 2) (Fig. 9D). Sexual dimorphic differences present in cephalothorax, urosome, antennule, P3 endopod, P5, P6 and caudal rami.

Cephalothorax (Fig. 9D), with posterolateral corniform processes less curved and developed than in the female.

Urosome (Figs. 9D, 14A) six-segmented, genital and first abdominal somites separated; first abdominal somite ornamented with stout ventral spinules posteriorly; post-genital somites without cuticular ridges dorsally.

Antennule (Fig. 14B) subchirocer, seven-segmented. First segment with one anterior protuberance ornamented with spinules, one row of outer spinules subdistally, and few inner spinules; with one plumose inner seta subdistally (lost during a dissecting process, but present in another paratype). Second segment elongate, with one row of spinules on outer bump, one transverse row of anterior spinules, one row of inner spinules, and one small anterior protuberance; with five inner setae (one uniplumose and four bare), three bare anterior setae, and one bare posterior seta. Third segment small, with seven bare inner setae. Fourth segment remarkably reduced as small sclerite, with two bare inner setae. Fifth segment swollen; inner margin ornamented with tiny spinules medially and several protuberances proximally, with one unispinulose spine; posterior surface with one uniplumose and seven bare setae near inner margin, and two long setae and one peduncle bearing one aesthetasc and one long bare seta near distal margin. Sixth segment small, slightly longer than wide, with three inner setae. Seventh segment tapering distally, slightly curved; inner margin with one bare seta; anterior surface with three bi-articulate setae; outer margin with three bi-articulate setae and one acrothek comprising one aesthetasc and two bare setae. Setal formula: 1-[1], 2-[9], 3-[7], 4-[2], 5-[11 + (1 + aesthetasc)], 6-[3], 7-[7 + acrothek].

P3 endopod (Fig. 14C) three-segmented; enp-1 as in female; enp-2 elongate, ornamented with one stout outer spinule and one row of inner spinules, distal margin produced into curved apophysis; enp-3 elongate, about four times as long as wide, apically with two plumose setae.

P5 (Figs. 14A, 14D) exopod and baseoendopod fused, forming single lobe; outer setophore elongate with one long seta; anterior margin with one row of long setules, two groups of spinules, and two tube pores; ventral surface with one tube pore (next to endopodal seta) and one row of subdistal spinules; posterior margin with one fishbone-like seta (representing endopod) and few setules; distal margin with one fishbone-like and one plumose seta subapically, and one unipinnate seta apically; dorsal surface with few long setules proximally.

P6 (Fig. 14A) represented by a single plate, unarmed; both sides with a transverse gap.

Caudal rami (Figs. 9D, 14A) parallel, slightly shorter than those of females, about four times as long wide.

Etymology. The epithet is derived from the Latin noun *comptus* meaning “hair accessory” and refers to ornamentations on the third segment of the female antennule and the ventral surface of the second abdominal somite in both sexes. It is in the nominative singular; gender masculine.

Remarks. As mentioned above in the remarks regarding *B*. *lacuna* sp. nov., the new species resembles most *B*. *lacuna* sp. nov. in terms of general body shape and mouthpart appendages. However, morphological differences are present in the ornamentation of the anal operculum, shape of the female genital double-somite, length/width ratio of the caudal rami, and sexually dimorphic features in the male P3. Detailed morphological findings can also support this separation: the dorsal protrusion on the first free prosomite has four sensillum-bearing socles in *B. comptus* sp. nov. (*vs*. six socles in *B*. *lacuna* sp. nov.), and the first post-genital somite is armed with stout ventral spinules (*vs*. ornamentation comprising fine spinules in *B*. *lacuna* sp. nov.).

*Bicorniphontodes huysi* sp. nov.

urn:lsid:zoobank.org:act:C2AF1702-965F-41EC-8AAD-BC54F13A69AE

Figures 15–20

**Figure 15:**
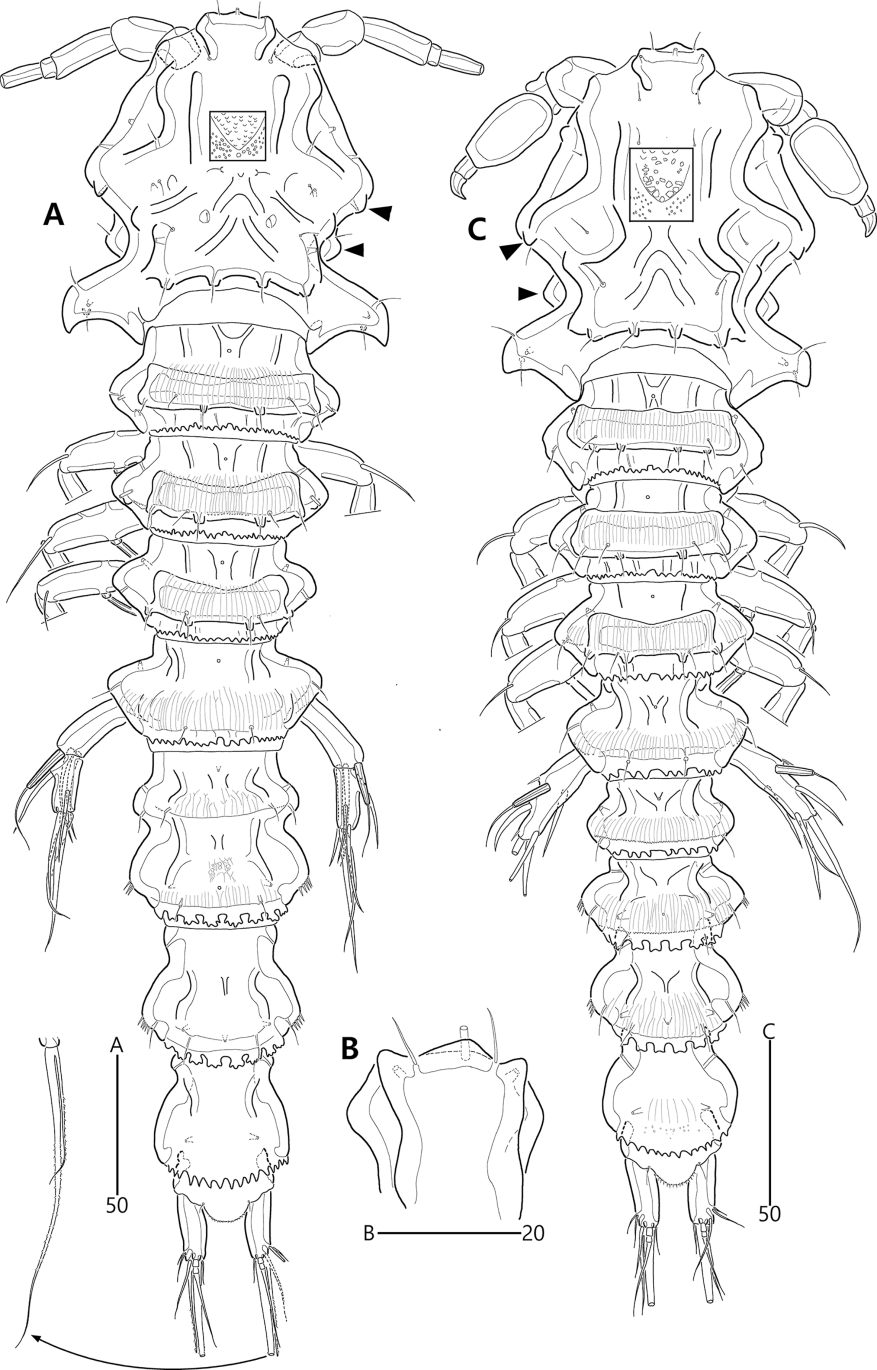
*Bicorniphontodes huysi* sp. nov., female, holotype (A–B), male, paratype 1 (C). (A) Female habitus, dorsal; (B) Rostrum, ventral; (C) Male habitus, dorsal. Arrowheads indicate apomorphies of *B*. *huysi* sp. nov.

**Figure 16:**
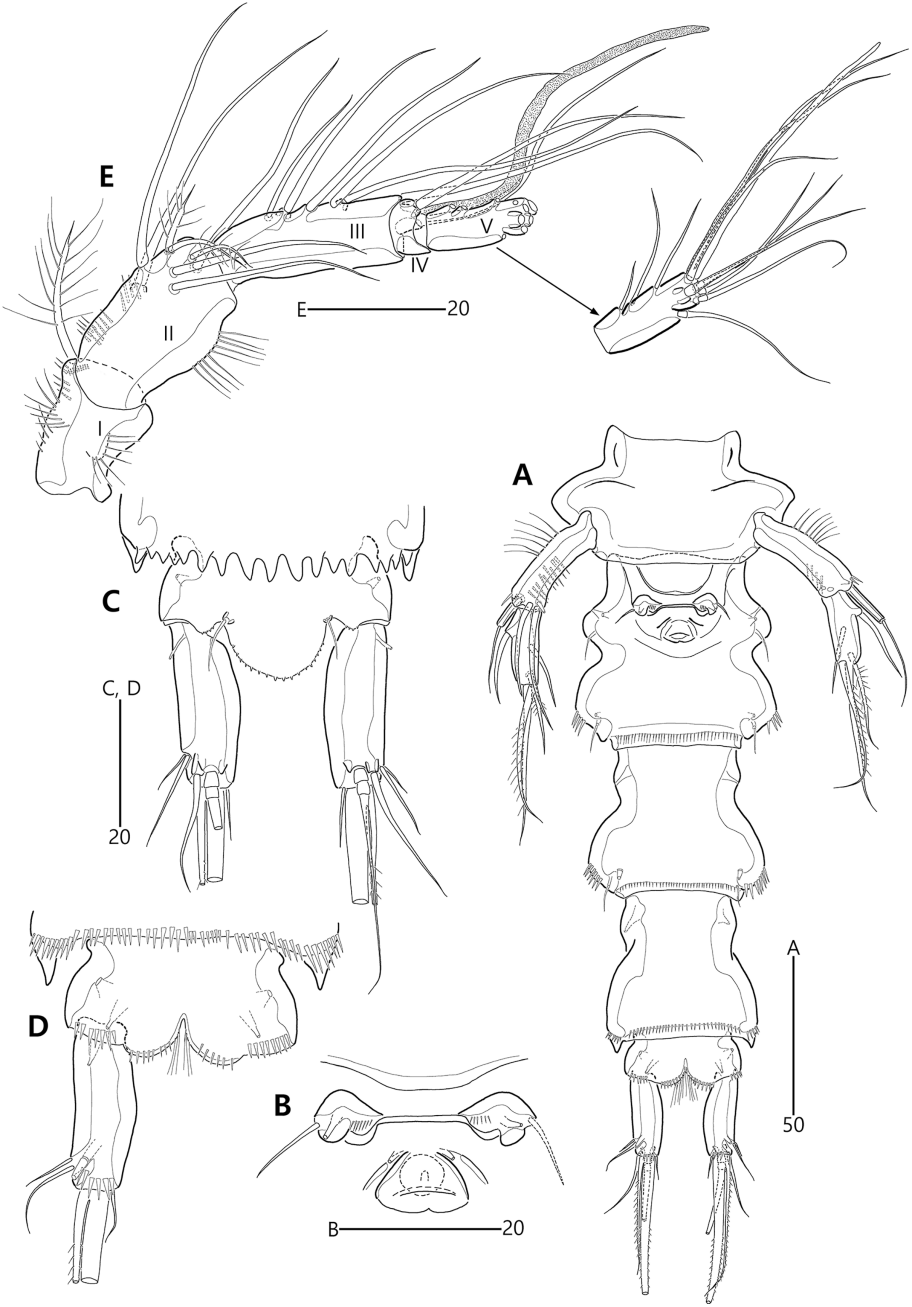
*Bicorniphontodes huysi* sp. nov., female, holotype (A–E). (A) Urosome, ventral; (B) Genital field; (C) Anal somite and caudal rami, dorsal; (D) Anal somite and right caudal ramus, ventral; (E) Antennule.

**Figure 17:**
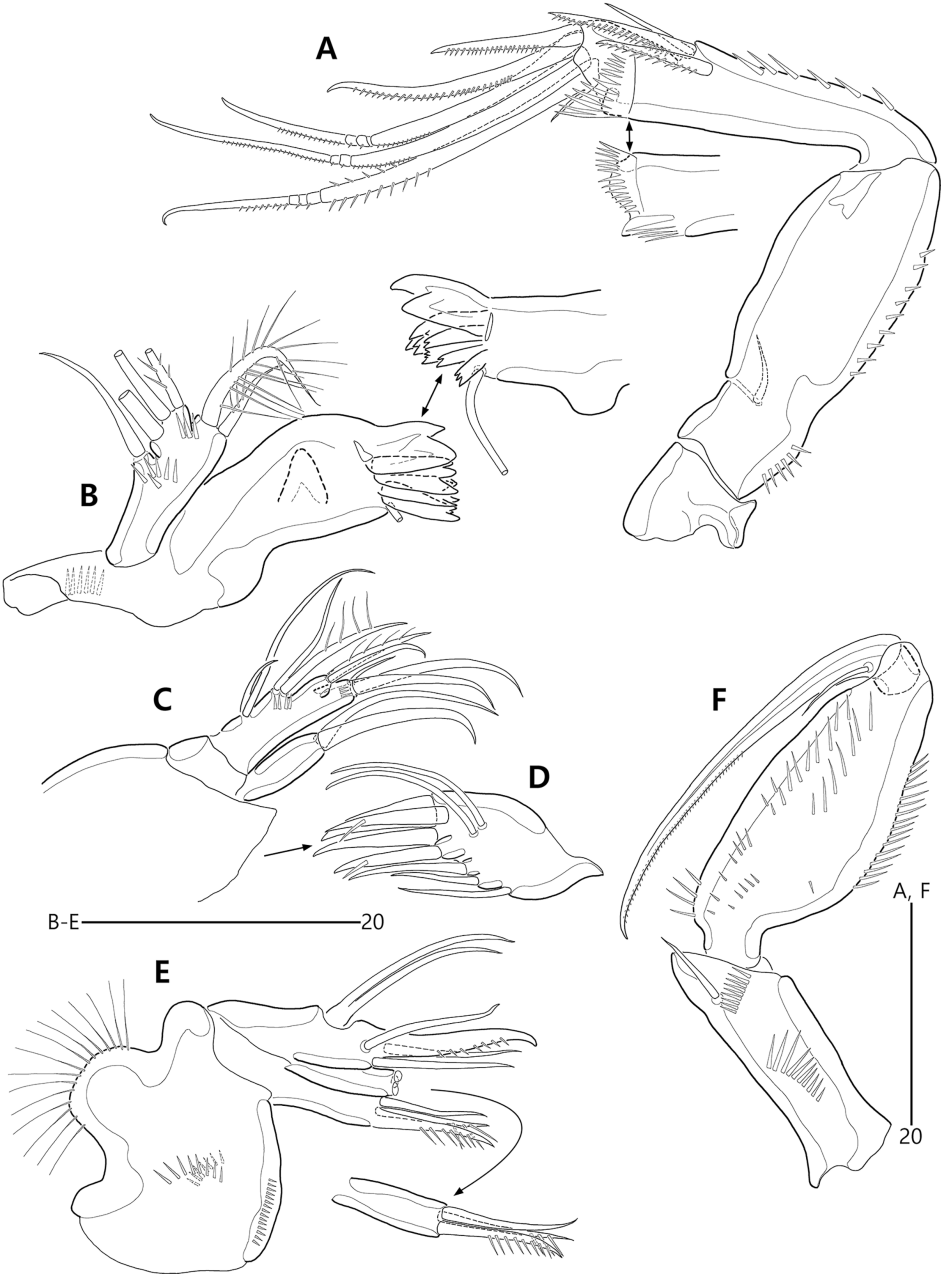
*Bicorniphontodes huysi* sp. nov., female, holotype (A–F). (A) Antenna; (B) Mandible; (C) Maxillule lacking its praecoxal arthrite; (D) Maxillular praecoxal arthrite; (E) Maxilla; (F) Maxilliped.

**Figure 18:**
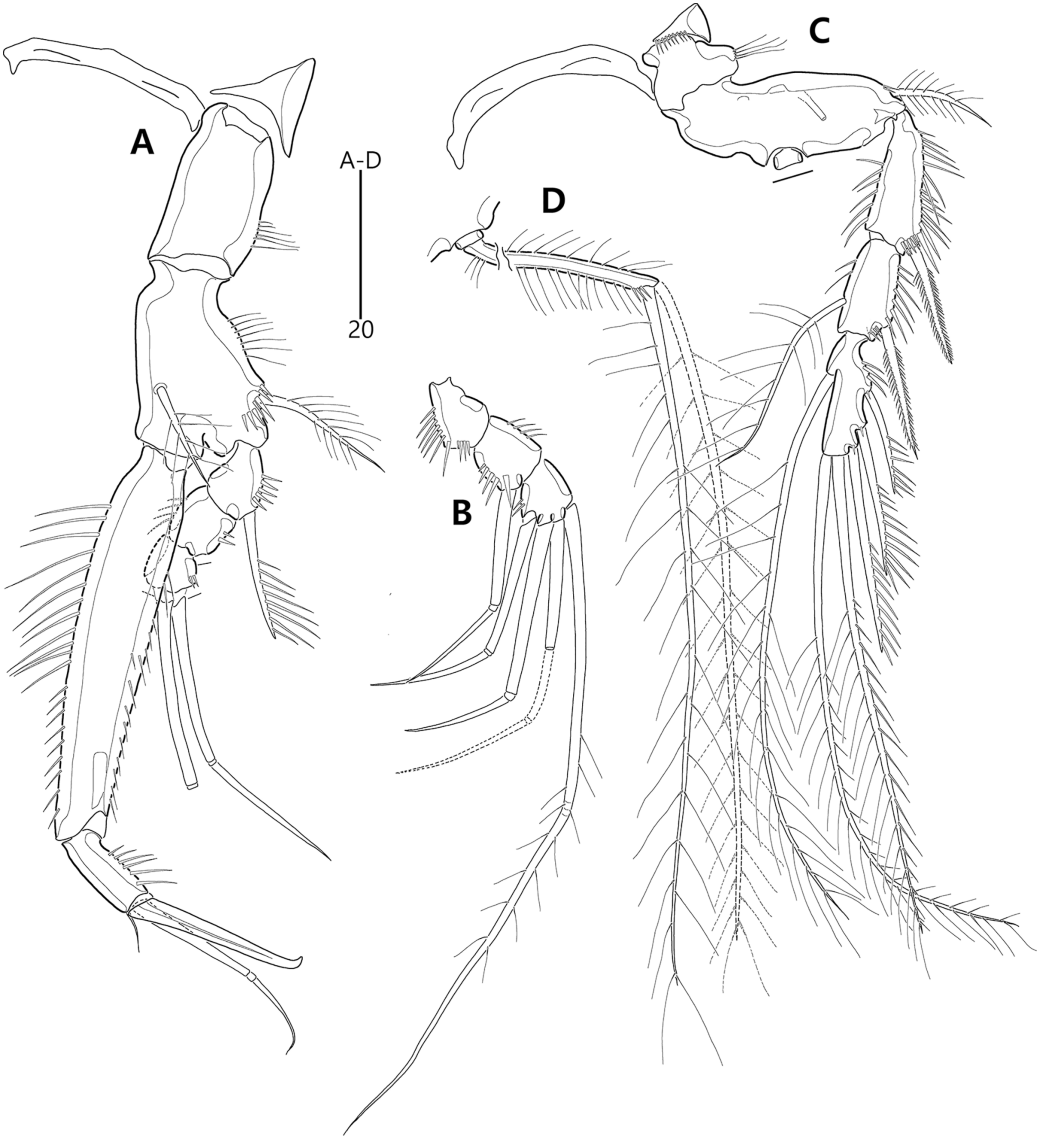
*Bicorniphontodes huysi* sp. nov., female, holotype (A–D). (A) P1, anterior; (B) P1 exopod, anterior; (C) P2 lacking its endopod, anterior; (D) P2 endopod, anterior.

**Figure 19:**
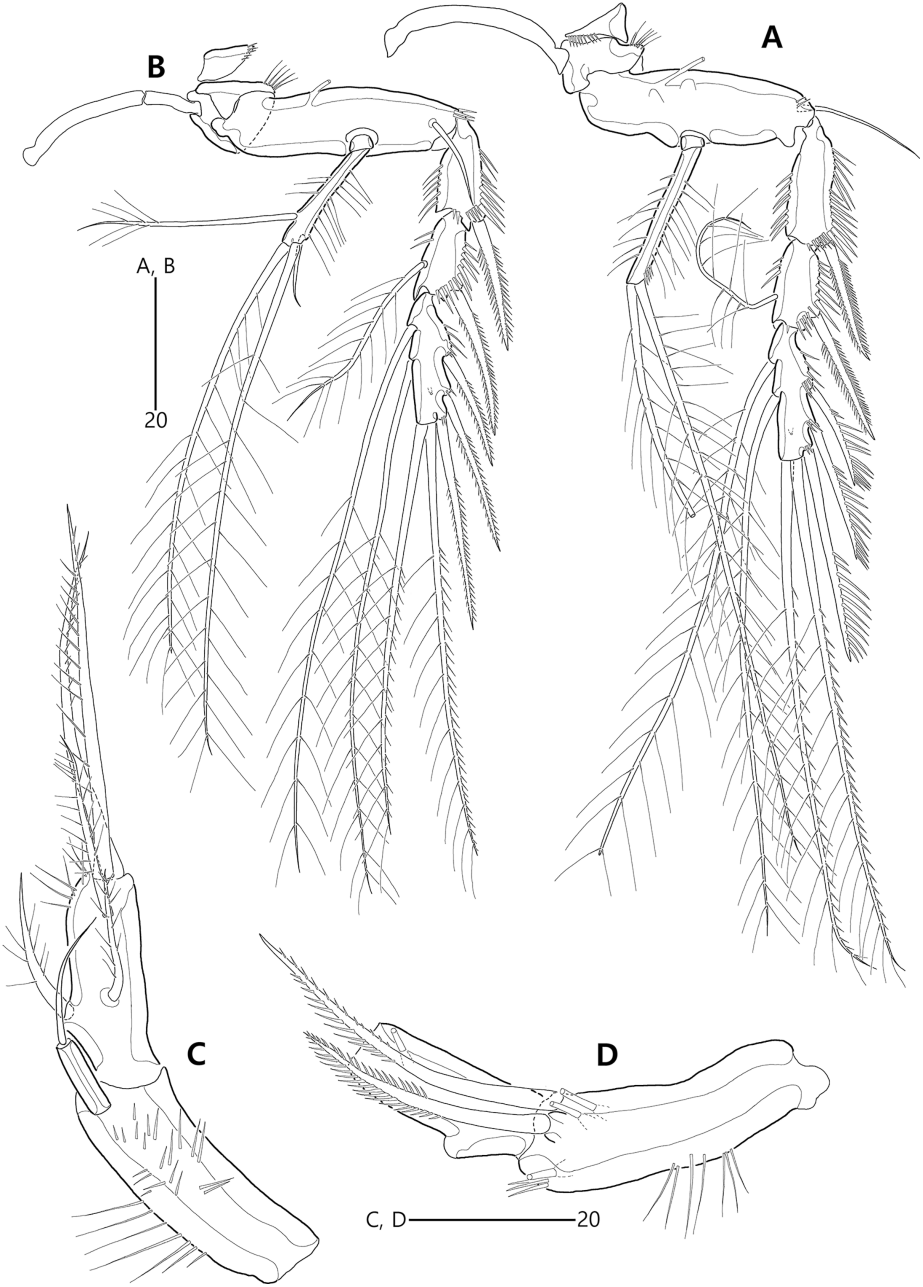
*Bicorniphontodes huysi* sp. nov., female, holotype (A–D). (A) P3, anterior; (B) P4, anterior; (C) P5, dorsal; (D) P5 baseoendopod and exopod omitted its setal armature, ventral.

**Figure 20:**
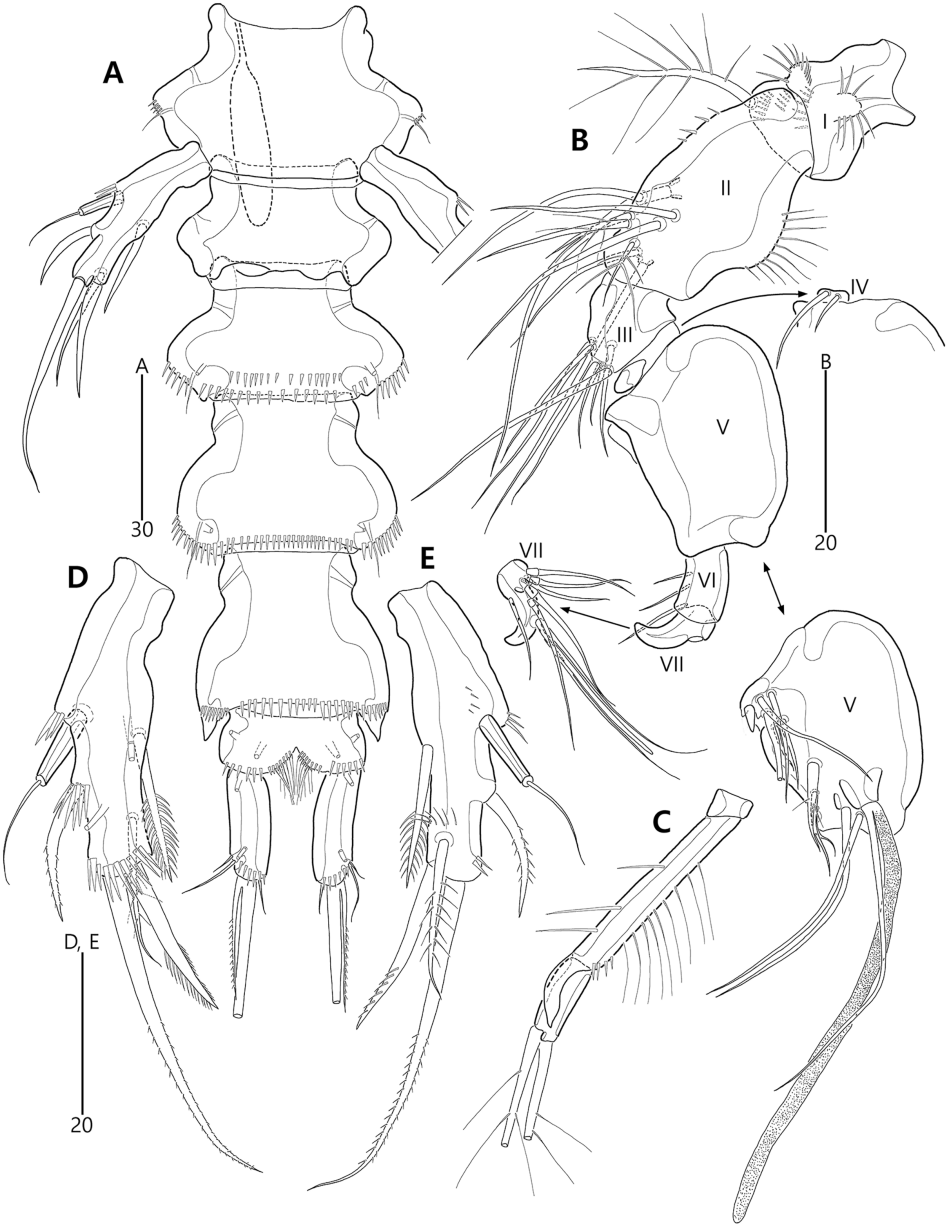
*Bicorniphontodes huysi* sp. nov., male, paratype 1 (A–E). (A) Urosome, ventral; (B) Antennule; (C) P3 endopod, anterior; (D) P5, ventral; (E) P5, dorsal.

*Laophontodes bicornis* Scott A., 1896: Kim, 2013, p. 51, Fig. 19.

Type material.—Holotype: ♀ dissected on 12 slides (MABIK CR00248418), Hagosudong beach of Udo Island where is a small island off Jejudo Island, Udo-myeon, Jeju-si, Jeju-do, South Korea, 33°30′49.9″N, 126°57′29.8″E, 0–1 m depth, sandy sediments, sampled by a hand net with 50 µm mesh, J.G. Kim leg., June 24, 2014. Paratypes: 1♂ dissected on 12 slides (paratype 1, MABIK CR00248419), 2♀♀ dissected on each slide (paratypes 2–3, MInRB-Hr74-S003–MInRB-Hr74-S004), 1♀, 1♂ preserved in a vial with 80% ethanol (paratypes 4–5, MInRB-Hr74-L001), collection data as holotype.

Description of female (based on holotype MABIK CR00248418).—Habitus (Fig. 15A) cylindrical, tapering posteriorly; total body length about 450 µm from tip of rostrum to posterior end of caudal rami in dorsal aspect (range = 399–450 µm; mean = 420 µm, *n* = 4).

Rostrum (Figs. 15A, 15B) prominent, basally fused to cephalic shield; apical tip less produced than in *B*. *comptus* sp. nov.; apically with one tube pore and two sensilla, and subapically with two pores.

Cephalothorax (Fig. 15A). Integument with more cuticular ridges than in the preceding two species, medially with V-shaped inner cuticular ridge; dorsoposterior ridge well-developed, with four sensillum-bearing socles; dorsal surface with dented ornamentation (see insert in Fig. 15A); mediolateral extensions with blunt tip and one small protrusion posteriorly (see upper arrowhead in Fig. 15A); posterolateral corniform processes weakly curved backwardly, less developed than in the preceding two species; additional lateral projections present between mediolateral extensions and posterolateral corniform processes (see lower arrowhead in Fig. 15A). Pedigerous prosomites extended laterally as in the preceding two species; dorsal surface anteriorly with one median and two lateral cuticular ridges, one median pore, and one pair of lateral pores (absent in P2-bearing somite), and posteriorly with sclerotized protrusion carrying two subdistal sensilla and four distal sensillum-bearing socles; dorsolateral surface with one pair of tube pores in P2–P3-bearing somites; lateral margin with one pair of lateral pores in P2- and P4-bearing somites; hyaline frills crenate.

P5-bearing somite (Figs. 15A, 16A) inverse trapezoidal in dorsal aspect as in the preceding two species; dorsal surface anteriorly with one median pore, one pair of lateral pores and cuticular ridges, and posteriorly with one pair of sensilla; lateral margin with one pair of sensilla; hyaline frill crenate. Genital double-somite completely fused, original division indicated by lateral contraction; dorsal aspect of original genital somite with one medial pore anteriorly, cuticular ridges medially, one pair of pores laterally, and four sensilla posteriorly; ventral aspect of original genital somite with one pair of ventrolateral pores; original first abdominal somite dorsally with vertical ridges, three pores, and two sensilla, laterally with one pair of spinular rows, ventrally with one pair of lateral pores, one pair of lateral sensilla, and one row of fine posterior spinules; hyaline frill composed of eight distinct projections with 1–4 lappets. Genital field (Figs. 16A, 16B) with transverse genital slit formed by fusion of both gonopores, covered by separate operculum bearing one bare seta, one tube pore, and one row of fine spinules (vestigial P6); copulatory pore large, posterior to genital slit, accompanied by one pair of tube pores. Second abdominal somite elongate, posteriorly expanded; lateral margins with one pair of pores, one pair of spinular rows, and one pair of sensilla; dorsal surface with cuticular ridges, three pores, and one pair of sensilla; ventral ornamentation and hyaline frill as in preceding somite. Penultimate somite slightly narrower than preceding somite; laterally with one pair of anterior pores; dorsally with cuticular ridges and two pores; both sides of posterior margin distinctly produced, each with one row of ventral spinules; ventral surface with spinules subdistally; hyaline frill serrate. Anal somite (Figs. 15A, 16A, 16C, 16D) small, with one pair of dorsolateral pores, one pair of ventral pores, and one pair of ventral tube pores; semicircle operculum distinctly prominent, ornamented with minute processes, and accompanied by one pair of sensilla basally; pedestal for insertions of caudal rami ventrally with spinules; medial cleft weakly developed, ornamented with bunches of setules and row of spinules.

Caudal rami (Figs. 15A, 16A, 16C, 16D) remarkably shorter than those of preceding two species, slightly longer than anal somite, about three times as long as wide; medial margin slightly convex; ornamentation and setal position as in the preceding species; seta II about twice as long as seta I; seta III slightly shorter than caudal ramus, about 1.9 times longer than seta II; seta V shorter than those of the preceding two species, about twice as long as seta IV; seta VI slightly longer than seta I; tri-articulate seta VII about 1.5 times longer than seta III.

Antennule (Fig. 16E) five-segmented. First segment with one row of anterior spinules on bump near outer margin, three rows of inner spinules, and one plumose inner seta bearing set of minute spinules at its base. Second segment shorter than those of the preceding two species; outer margin with weak bump bearing a setular row; inner margin with three rows of spinules posteriorly; with two plumose and two bare inner setae, one plumose and two bare anterior setae, and one bare posterior seta; anterior surface without the small protuberance that is present in the preceding two species. Third segment shorter than in the preceding two species, about 2.7 times as long as wide, unornamented; inner margin with five bare setae medially and one bare seta subdistally; distal margin ventrally with peduncle bearing one aesthetasc and one long bare seta (both fused basally). Fourth segment small, with one long bare inner seta. Distal segment elongate; inner margin with three bare setae; outer margin with one bi-articulate seta subdistally; anterior surface with one bi-articulate and one bare seta subdistally; distal margin with one inner acrothek composed of one aesthetasc and two long setae, and with three bi-articulate outer setae. Setal formula: 1-[1], 2-[8], 3-[6 + (1 + aesthetasc)], 4-[1], 5-[9 + acrothek].

Antenna (Fig. 17A) three-segmented. Coxa small, unornamented. Allobasis elongate, with two rows of abexopodal spinules; scar of original division between basis and first endopodal segment indicated by small crack. Exopod represented by one small bare seta. Free endopodal segment elongate, with one row of abexopodal spinules and two subdistal spinulose frills; lateral armature composed of two distinctly unipinnate spines and one bare seta; distal armature composed of two distinctly pinnate spines, three geniculate setae (innermost one bipinnate proximally, unipinnate distally, and basally fused to tiny seta), and one tiny seta.

Mandible (Fig. 17B). Coxa with one bulge medially and one row of spinules proximally; gnathobase armed with multicuspidate teeth and one seta. Mandibular palp with two rows of spinules; original basis distally with one long plumose and one short uniplumose seta; exopod small, incorporated into basis, with one bare seta distally; endopod larger than exopod, incorporated into basis, with three setae distally.

Maxillule (Figs. 17C, 17D). Praecoxa unornamented; arthrite (came off during dissection process) well-developed, distally with two bare posterior spines and two anterior spines bearing one spinule, laterally with three bare setae, and anteriorly with two juxtaposed setae. Coxal endite cylindrical, with two bare distal setae. Basis, exopod and endopod fused. Basis with two rows of spinules anteriorly, one small and two long bare setae distally, and one uniplumose and one bare seta subdistally. Exopod represented by a set of one small and one long seta. Endopod represented by a set of one bare and one uniplumose seta.

Maxilla (Fig. 17E). Syncoxa with long setules along outer bump, one row of inner spinules, one row of anterior spinules, and one row of posterior spinules; with two endites: proximal endite distally with one bare seta, two unipinnate setae (proximal one fused to endite basally); distal endite with one bare seta and two unipinnate setae. Allobasis drawn out into stout unipinnate claw, with three accompanying bare setae. Endopod incorporated into basis, represented by a set of two bare setae (fused basally).

Maxilliped (Fig. 17F). Syncoxa elongate, with two rows of spinules medially and one short bare seta subdistally. Basis more densely ornamented than in the preceding two species; inner margin with one row of spinules proximally; median surface with moderate and short spinules; outer margin convex, with one row of spinules. Endopod small, one-segmented, with one stout unipinnate claw slightly beyond basis, carrying one tiny seta.

P1 (Figs. 18A, 18B). Praecoxa triangular, unornamented, smaller than in the preceding two species. Intercoxal sclerite transversely elongate. Coxa about 1.6 times as long as wide, with one row of outer setules. Basis with one row of outer setules and one row of anterior spinules (near insertion of outer seta), with distinct pedestal for insertion of inner ramus; outer seta plumose; plumose inner seta inserted on anterior margin near inner margin. Exopod three-segmented, reaching to proximal third of enp-1; exp-1 with one row of outer spinules, one row of anterior spinules at base of outer spine, with one unipinnate and comb-like outer spine; exp-2 with one row of outer spinules, one large anterior spinule at base of outer seta, one row of inner spinules, and one geniculate outer seta; exp-3 small, with few outer spinules and four geniculate setae (of which innermost one longest and plumose distally). Endopod prehensile, two-segmented; enp-1 elongate, about 2.3 times longer than exopod, with one inner row of long setules proximally and one inner row of short spinules distally, and one outer row of spinules; enp-2 distinctly smaller than in preceding segment, about 2.5 times as long as wide, with one row of outer spinules, distally with one stout spine, one geniculate and one tiny seta.

P2–P4 (Figs. 18C, 18D, 19A, 19B). Praecoxae small, distally with spinules. Intercoxal sclerite transversely elongate. Coxae with a set of outer setules. Bases transversely elongate, with one plumose (in P2) or bare (in P3–P4) outer seta accompanied by few spinules at its base; P2 with one anterior tube pore, P3–P4 with one outer tube pore. Exopods three-segmented; exp-1 elongate, with two outer rows of spinules, one inner row of spinules, and few spinules at base of outer spine, and with one pinnate outer spine; exp-2 smallest, with one outer row of spinules, one inner row of spinules, and one anterior row of spinules near base of outer spine, and with one pinnate outer spine and one plumose inner seta; P2 exp-2 with, P3–P4 exp-2 without one anterior pore; exp-3 elongate, with one outer row of spinules proximally, three outer spines (unipinnate and comb-like in P2–P3, and bipinnate in P4) carrying few anterior spinules at its base, two distal setae (ornamented with outer spinules and inner setules), and one (in P2) or two (in P3–P4) plumose inner setae; P2 exp-3 without, P3–P4 exp-3 with one anterior pore. Endopods two-segmented; enp-1 small, unornamented; enp-2 elongate, ornamented with inner and outer setules; P2–P3 enp-2 with one additional group of outer spinules subdistally, and with two plumose setae distally; P4 enp-2 with one bare outer seta (distinctly shorter than those of the preceding two species), two plumose distal setae, and one plumose inner seta.

P5 (Figs. 16A, 19C, 19D) two-segmented as in the preceding two species. Baseoendopod elongate; anterior margin with one row of long setules medially, and one tube pore and one row of spinules subdistally; dorsal surface ornamented with spinules; ventral surface subdistally with two fishbone-like setae (originally arising from vestigial endopodal lobe) and two tube pores; outer setophore shorter than those of the preceding two species, with one bare seta distally. Exopod about 0.8 times as long as baseoendopod; anterior margin with setules subdistally and one plumose seta proximally; dorsal surface with two plumose setae; distal margin with two long unipinnate setae; ventral surface with one tube pore subdistally.

Description of male (based on paratype 1 MABIK CR00248419). Total body length smaller than female, about 362 µm (range = 350–362 µm; mean = 356 µm, *n* = 2) (Fig. 15C). Sexual dimorphic differences detected in cephalothorax, urosome, antennule, P3 endopod, P5 and P6.

Cephalothorax (Fig. 15C), posterolateral corniform processes less developed and curved than in the female.

Urosome (Figs. 15C, 20A) six-segmented, genital and first abdominal somites separated; genital somite smaller than first abdominal somite, with crenate hyaline frill; first abdominal somite with one pair of lateral pores anteriorly and two rows of ventral spinules posteriorly.

Antennule (Fig. 20B) subchirocer, seven-segmented. First segment with one row of spinules along with small anterior bump, one row of inner spinules, and three rows of posterior spinules; with one plumose inner seta subdistally. Second segment longest, with one row of minute inner spinules proximally, and one row of spinules on outer bump medially; with one plumose and two bare inner setae, one plumose and two bare anterior setae, and three bare posterior setae. Third segment small, with two delicate and four long bare inner setae, and one bare ventral seta. Fourth segment minute, with two setae. Fifth segment swollen; inner margin with one small spine proximally; posterior surface proximally with five bare setae, medially with one plumose and two bare setae, and subdistally with two long bare setae and one distinct peduncle for one long bare seta and one aesthetasc (both elements fused basally); proximal part of inner margin with fewer protrusions than in the preceding two species. Sixth segment small, with three inner setae posteriorly. Seventh segment tapering distally, with one bare inner seta, two bi-articulate anterior setae, three bi-articulate outer setae, and one outer acrothek composed of one aesthetasc and two bare setae. Setal formula: 1-[1], 2-[9], 3-[7], 4-[2], 5-[11 + (1 + aesthetasc)], 6-[3], 7-[6 + acrothek].

P3 with three-segmented endopod (Fig. 20C); enp-1 small, unornamented; enp-2 elongate, with few stout outer spinules, one row of inner setules medially, and one row of inner spinules subdistally; distal margin with one recurved apophysis; enp-3 about 2.6 times as long as wide, unornamented, with two plumose distal setae.

P5 exopod fused to baseoendopod forming single lobe as in the preceding two species (Figs. 19D, 19E); anterior margin with three groups of spinules and one tube pore; ventral surface with one tube pore medially and one row of spinules subdistally; dorsal surface with two groups of spinules; endopodal lobe represented by one fishbone-like seta; outer setophore with one long seta; original exopod with one weakly pinnate anterior seta, one plumose dorsal seta, and one small fishbone-like and one long weakly pinnate distal seta.

P6 (Fig. 20A) vestigial, asymmetrical, unarmed; left leg probably fused to somite.

Etymology.—The species is named in recognition of Professor Rony Huys (The Natural History Museum) for his major contributions to the taxonomy of harpacticoid copepods. It is a noun in the genitive case; gender masculine.

Remarks. Kim (2013) first reported the occurrence of *Bicorniphontodes bicornis* (known as *Laophontodes bicornis* at the time) in Korean waters based on materials collected from Seoqwipo, Jeju Island; that work briefly mentioned that this species was also present in the coralline sands of Udo Islet (northeast of Jeju Island). In a revision of the family Ancorabolidae, Lee & Huys (2019) recognized that this Korean species considerably differs from the complete description of *B*. *bicornis* provided by George & Gheerardyn (2015) in terms of cephalothorax posterolateral corniform processes, body somites, P2–P4 endopodal segments, length of the caudal rami, and male antennule (cf. Lee & Huys, 2019). They suggested that Kim’s material constituted a new species, although they did not address its taxonomic position.

During taxonomic studies on Korean harpacticoids, we collected three *Bicorniphontodes* species: *B*. *lacuna* sp. nov., *B*. *bicornis sensu* Kim, 2013, and *Bicorniphontodes* sp. (the present study did not examine the latter species because only two female specimens were collected) from two sandy beaches on Udo Islet near Jeju Island. Among them, several female specimens were identified as *B*. *bicornis sensu* Kim, 2013 because they concurred with Kim’s photographs (*2013*: Fig. 19) of the habitus, urosome and caudal rami: the posterolateral corniform processes on the cephalothorax are less developed (short and blunt); the P5-bearing somite is strongly produced laterally; and the caudal ramus is distinctly shorter than the penultimate somite. These findings imply that our specimens can be considered conspecific with Kim’s material. However, detailed examination of specimens from Udo Islet revealed that they do not match Kim’s description. In particular, the length of the caudal rami is approximately three times greater than its width (*vs*. 4–5 times), the mandibular palp consists of six setae (*vs*. five setae), the P2–P4 endopods are two-segmented (*vs*. one-segmented), and the male antennule is seven-segmented (the small fourth segment was presumably overlooked). These observations suggest that Kim (2013) misjudged the morphology of the Jeju Island materials. Thus, we decided to clarify the taxonomic assignment of *B*. *bicornis sensu* Kim, 2013 based on specimens from Udo Islet.

The Udo Islet specimens, herein named *B*. *huysi* sp. nov., are similar to *B*. *hamatus*, in terms of cephalothorax and shape of the caudal rami. *Bicorniphontodes hamatus* was first reported from a New Zealand harbor by Thomson (1883); the original description contained several errors in terms of female antennules, P1 and P5. Subsequently, this species was mentioned by Lang (1948), who reevaluated its thoracic leg setal armature based on other New Zealand material. This limited information regarding *B*. *hamatus* has caused taxonomic confusion (Lee & Huys, 2019), which led us to avoid comparison with our new species. Nevertheless, *B*. *huysi* sp. nov. can be distinguished from the New Zealand species by the length/width ratio of the urosomites. For example, the first abdominal somite of the new species is more wider than the genital somite, however *B*. *hamatus* has reversed trapezoidal genital double-somite. The length/width ratio of the penultimate somites are significantly different (0.9 in *B*. *huysi* sp. nov. *vs*. 0.6 in *B*. *hamatus*).

**Cladistic analysis based on morphological characters**

For the phylogenetic analysis of the species of *Bicorniphontodes*, a character matrix was made by scoring morphological characters as binary (0–1) or multistate (0–n). Micro-characters (*e.g*., surface structures and integument ornamentation) were included among the 41 chosen characters, considering recent studies that revealed their utility during morphology-based phylogenetic analyses of harpacticoid copepods (*e.g*., Karanovic & Lee, 2012; Karanovic & Kim, 2014). Some characters were adopted from the previous phylogenetic study of *Bicorniphontodes* (George, Glatzel & Schröder, 2019). The 41 selected characters are listed below (0 = plesiomorphic state, 1–2 = succeeding apomorphic states):

0. Rostrum with a subapical tube pore (0); absent (1).

1. Rostrum with moderate apical tip (0); distinctly projected apical tip (1).

2. All free body somites with moderate hyaline frill (0); serrate or crenate (1).

3. Cephalothorax, mediolateral extensions, absent (0); present (1).

4. Cephalothorax, posterolateral corniform processes, absent (0); present (1).

5. Cephalothorax, mediolateral extensions with a rounded tip (0); a cuspidate tip (1).

6. Cephalothorax, mediolateral extensions without protuberances (0); with 1 posterior protuberance at least in ♂ (1); with 2 protuberances (2).

7. Cephalothorax, posterolateral corniform processes, less developed or short and blunt (0); recurved backwardly (1); distinctly developed (2).

8. Cephalothorax, posterolateral corniform processes with smooth lateral margin (0); with a subdistal notch (1).

9. Cephalothorax with a smooth dorsal surface (0); with dorsal cuticular ridges (1).

10. Cephalothorax without any additional lateral projections (0); with small lateral projections between mediolateral extensions and posterolateral processes (1).

11. Free prosomites with a smooth dorsal surface (0); with a dorsal sclerotized area (1).

12. Urosomites with a smooth dorsal surface (0); with dorsal cuticular ridges (1).

13. Genital double-somite with smooth lateral contraction (0); with a pair of distinct lateral furrows (1).

14. First and second abdominal somites with serrate or crenate hyaline frills (0); with distinct projections carrying lappets (1).

15. Caudal rami without sexual dimorphism (0); distinctly shorter, parallel in ♂ (1).

16. Caudal rami, length/width ratio less than 4.5 times (0); more than 4.5 times (1).

17. Caudal setae I and II arising centrally from the outer margin (0); displaced subapically (1).

18. Antennule, 2^nd^ segment without any anterior protrusions (0); with an anterior protrusion in both sexes (1).

19. Antennule, 3^rd^ segment without any ornamentation (0); with a spinular row on the anterior margin in ♀ (1).

20. Antennule ♂ subchirocer (0); chirocer (1).

21. Antennary endopod, lateral armature with 3 elements (0); with 2 elements (1).

22. Antennary endopod, distal armature with 6 elements (0); with 5 elements (a tiny seta or a tube pore absent) (1).

23. Mandibular palp with 6 setae (0); with < 6 setae (1).

24. Mandibular gnathobase with a seta (0); without setae (1).

25. Maxillule, exopod represented by 2 setae (0); by 1 seta (1).

26. Maxillule, endopod represented by 2 setae (0); by 1 seta (1).

27. Maxilla, endopod 1-segmented (0); endopod incorporated into basis (1).

28. Maxilla, coxal endite with 3 elements (0); with 2 elements (1).

29. Maxilliped, syncoxa with 1 subdistal seta (0); unarmed (1).

30. P1 exopod ornamented with outer spinules in all segments (0); unornamented segment present (1).

31. P1 enp-2, distal seta non-geniculate (0); geniculate (1).

32. P2–P3 exp-3, outer spines pinnate (0); unipinnate and comb-like (1).

33. P2–P4 coxae, a set of outer setules present (0); absent (1).

34. P3 enp-2 ♀ with an outer element (0); absent (1).

35. P3 enp-2 ♂ with an apophysis (0); with a tube pore (1).

36. P4 enp-2, outer element elongation (0); outer element distinctly reduced in length (1).

37. P5 ♀ 2-segmented (0); 1-segmented (1).

38. P5 exopod ♀ with 1 tube pore (0); absent (1).

39. P5 baseoendopod ♀ with 2 tube pores on ventral surface (0); with 1 tube pore (1); absent (2).

40. P5 baseoendopod ♂ with 2 tube pores near endopodal element (0); with 1 tube pore (1); absent (2).

**Figure 21:**
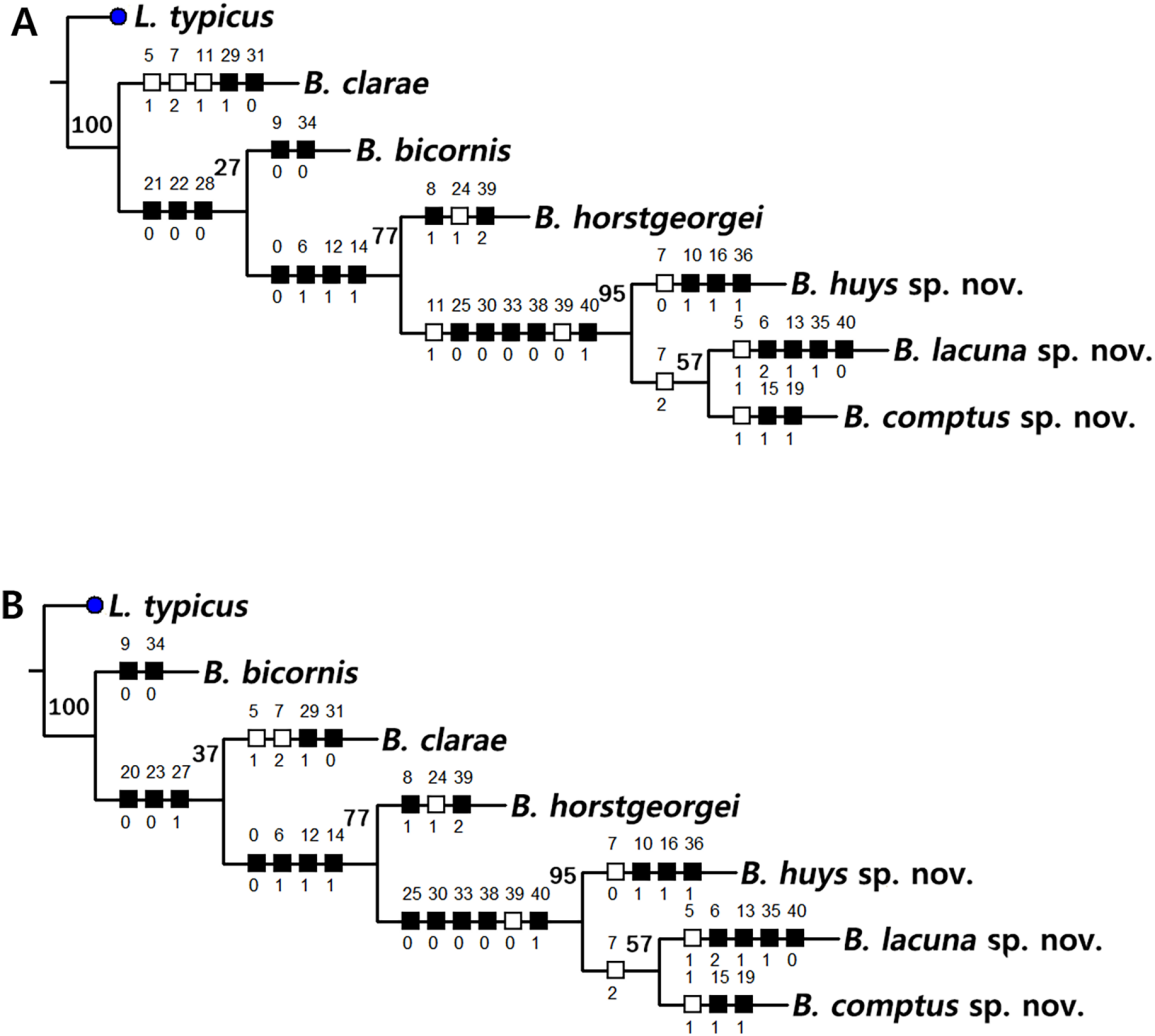
Two most parsimonious cladograms of *Bicorniphontodes* species, based on the 41 selected morphological states. Filled squares, presumed synapomorphies or autapomorphies; open squares, presumed homoplasies; numbers above squares, the number of characters; numbers below squares, characters states; large numbers on branches, bootstrap values.

Based on the data matrix shown in Table 1, the phylogenetic analysis of *Bicorniphontodes* species, with *L*. *typicus* as the outgroup, was performed using NONA software (Goloboff, 1999). Ratchet Island Hopper analysis of equally weighted characters yielded two most parsimonious cladograms with a length of 59 steps, a consistency index (Ci) of 0.76, and a retention index (Ri) of 0.63 (Fig. 21). The values of Ci and Ri indices were generally low compared with previous phylogenetic studies of benthic harpacticoids that showed high rates of convergence within the taxon group (*e.g*., Karanovic & Hancock, 2009; Karanovic, Kim & Lee, 2015; Corgosinho et al., 2017). In both most parsimonious cladograms, the monophyly of *Bicorniphontodes* was supported, with a 100% bootstrap value indicating the well-defined ingroup by five unambiguous synapomorphies (Table 1): hyaline frills of free body somites are serrate or crenate (character no. 2: 1); cephalothorax has mediolateral extensions (character no. 3: 1); cephalothorax has posterolateral corniform processes (character no. 4: 1); caudal setae I and II are displaced subapically (character no. 17: 1); and the outer spines on the P2–P3 are unipinnate and comb-like (character 32: 1).

**Table 1:** Morphological data matrix for cladistic analysis of five *Bicorniphontodes* species and one outgroup taxon *Laophontodes typicus*.

Taxon	Characters				
		1	2	3	4
	0123456789	0123456789	0123456789	0123456789	0
*L*. *typicus*	1000000001	0000000010	1111110010	1101100110	2
*B*. *bicornis*	1111100100	0000000100	10010??000	1111000011	2
*B*. *clarae*	1111110201	0100000100	0110011111	1011100011	2
*B*. *horstgeorgei*	0011101111	0010100110	0000111100	1111100012	2
*B*. *lacuna* sp. nov.	0011112201	0111100110	0000000100	0110110000	0
*B*. *comptus* sp. nov.	0111101201	0110110111	0000000100	0110100000	1
*B*. *huysi* sp. nov.	0011101001	1110101100	0000000100	0110101000	1

**Note:**

0 = presumed plesiomorphic state; 1–2 = presumed apomorphy; ? = unknown state.

The topologies of the two cladograms are the same except for the species in a basal position (Figs. 21A, 21B). The first cladogram (Fig. 21A) implies that the most plesiomorphic species is *B*. *clarae* with two autapomorphies: the maxillipedal syncoxa is unarmed (character no. 29: 1); and the inner element on the distal margin of the P1 enp-2 is non-geniculate (character no. 31: 0). However, the basal position in the second cladogram (Fig. 21B) is *B*. *bicornis* with two autapomorphies: the cephalothorax has a smooth dorsal surface (character no. 9: 0); and the P3 enp-2 has an outer element (character no. 34: 0). A sister-group relationship of *B*. *clarae* with the remaining four species (*B*. *horstgeorgei*, *B*. *huysi* sp. nov., *B*. *lacuna* sp. nov. and *B*. *comptus* sp. nov.) is supported with more high bootstrap value (37%) than *B*. *bicornis* (27%) although both bootstraps of the basal node were lower than 50%. In both cladograms, *B*. *horstgeorgei* (from the Fiji Islands in the Pacific) was more closely aligned with the three new Korean species, supported by its moderate bootstrap value (77%). These four species shared four synapomorphies: the rostrum has a subapical tube pore (character no. 0: 0); the mediolateral extensions on the cephalothorax have protuberances on its tip (character no. 6: 1); the dorsal surfaces of urosomites have cuticular ridges (character no. 12: 1); and the first and second abdominal somites have distinct hyaline frills that are composed of projections carrying lappets (character no. 14: 1). The three new species from Korean waters were clustered together, supported by a high bootstrap value (95%), based on five synapomorphies: the maxillular exopod is represented by two setae (character no. 25: 0); all exopodal segments on the P1 are ornamented with outer spinules (character no. 30: 0); the coxae of the P2–P4 are ornamented with outer setules (character no. 33: 0); the exopod of the female P5 has a tube pore (character no. 38: 0); and the baseoendopod of the male P5 has a tube pore (character no. 40: 1).

In the Korean species clade, *B*. *huysi* sp. nov. was isolated from the *B*. *lacuna* sp. nov./*B*. *comptus* sp. nov. clade by three unambiguous autapomorphies: the cephalothorax has additional protrusions between mediolateral extrusions and posterolateral corniform processes (character no. 10: 1); the length/width ratio of the caudal rami is less than 4.5 (character no. 16: 1); and the outer element on the P4 enp-2 is distinctly reduced in length (character no. 36: 1). The sister-group relationship between *B*. *lacuna* sp. nov. and *B*. *comptus* sp. nov. was defined by only one homoplasy of the well-developed posterolateral corniform process on the cephalothorax (character no. 7: 2), which had a low bootstrap value (57%).

*Bicorniphontodes lacuna* sp. nov. can be distinguished from its sister taxon, *B*. *comptus* sp. nov., by the following four autapomorphies: mediolateral extensions on the cephalothorax have two small protrusions (character no. 6: 2); the division of the original genital double-somite division is marked by distinct lateral furrows (character no. 13: 1); the P3 enp-2 in the male has a tube pore (character no. 35: 1) rather than an apophysis, which is a general sexually dimorphic trait in ancorabolid copepods (see Discussion); and the baseoendopod of the male P5 has two tube pores near endopodal element (character no. 40: 0). The mediolateral extensions with a cuspidate tip (character no. 5: 1) are presumably homoplastic character states shown in *B. clarae* and *B. lacuna* sp. nov.

Two autapomorphies for *B*. *comptus* sp. nov. are present: the caudal rami show sexual dimorphism (they are divergent and elongated in the female, and parallel and relatively reduced in length in the male; character no. 15: 1) and the third segment of the female antennules has a row of inner spinules (character no. 19: 1). A homoplastic change, *i.e*., a pointed rostral tip (character no. 1: 1), is evident in this species.

## Discussion

In the frame of a revision of the genus *Laophontodes*, George, Glatzel & Schröder (2019) established the genus *Bicorniphontodes* for five species: *B*. *bicornis*, *B*. *clarae*, *B*. *hamatus*, *B*. *horstgeorgei*, and *B*. *ornatus*. They claimed that these species have a close relationship, supported by the following supposed synapomorphies: a pair of mediolateral triangular extensions and a pair of posterolateral corniform processes on the cephalothorax; crenate hyaline frills of body somites (with the exception of the anal somite); subdistal displacement of caudal setae I and II; the absence of an abexopodal seta on the antennary allobasis; and unipinnate and comb-like outer spines on the P2–P3 exp-3. Three new Korean species (*B*. *lacuna* sp. nov., *B*. *comptus* sp. nov., and *B*. *huysi* sp. nov.) also show these synapomorphies. Thus the allocation of these species to *Bicorniphontodes* is unproblematic.

The results of a parsimony analysis based on 41 morphological characters with *L*. *typicus* as outgroup and *B*. *hamatus* and *B*. *ornatus* excluded (Table 1; Figs. 21A, 21B), support the unambiguous synapomorphies (character nos. 2, 3, 4, 17, and 32) suggested by George, Glatzel & Schröder (2019). The presence/absence of antennary abexopodal seta was omitted from this analysis because the character state of *L*. *typicus* coincides with that of *Bicorniphontodes*, but there is no uncertainty regarding the morphological evidence of monophyly in this genus. With respect to the presence of posterolateral processes recurved backwardly on the cephalothorax (character no. 7), it was considered a synapomorphic trait (state = 1) in this cladistic analysis, but *B*. *huysi* sp. nov. has less developed processes (state = 0); more derived features (state = 2) are retained in *B*. *clarae*, *B*. *lacuna* sp. nov., and *B*. *comptus* sp. nov. The topology of our two cladistic trees implies that this character is secondarily evolved in *B*. *huysi* sp. nov.

Based on a morphological comparison, George, Glatzel & Schröder (2019) presumed that *B*. *bicornis* occupies the basal offshoot within the genus, considering the presence of plesiomorphic characters such as a lack of cuticular ridges on the cephalothorax and a small (segmented) maxillary endopod. This first divergence of *B*. *bicornis* was also reproduced in our second tree (see Fig. 21B) which has a very low bootstrap support of 37%. However, the position of *B*. *clarae* which is a basal taxon in our first tree (Fig. 21A) differs from the result of George, Glatzel & Schröder (2019) who suggested this species is the most terminal clade (see George, Glatzel & Schröder, 2019: Fig. 8). They suggested several autapomorphies for *B*. *clarae*: in the cephalothorax, the mediolateral extension tips are cuspidate (character no. 5: 1) and the posterolateral corniform processes are much more developed (character no. 7: 2); the P2–P4 tergites are sclerotized (character no. 11: 1) and exhibit several sensillum-bearing socles; the caudal rami are slender and slightly divergent (this character was unselected in our cladistic analysis); and the distal armature of the free antennary endopod has only five setae and lacks a very slim seta (character no. 22: 1). However, except for the last character of the antenna, some of these characteristic features are present in the three new species. Therefore, the remaining four features should be excluded. Our analysis suggested that the characters no. 5, 7 and 11 in *B*. *clarae* are clearly homoplastic and identified two autapomorphies for *B*. *clarae*: the syncoxal maxillipedal element is absent (character no. 29: 1) and the distal seta on the P1 enp-2 is non-geniculate (character no. 31: 0). As suggested by George (2020), the last autapomorphic state may result from reverse transformations.

The sister-group relationship between *B*. *horstgeorgei* and the clade formed by the three Korean species was recovered in our parsimony analysis, based on the four synapomorphies: the rostrum has a subdistal median dorsal tube pore (character no. 0: 0); mediolateral extensions of the cephalothorax have a protuberance at least in the male (character no. 6: 1); urosomites have dorsal ridges (character no. 12: 1); and hyaline frills of the first and second abdominal somites are ornamented with distinct projections carrying lappets (character no. 14: 1). The apomorphic state of character no. 14 was shared with *B*. *ornatus* (see Krishnaswamy, 1957: Fig. 15), which was excluded from our cladistic analysis because its description lacks the required morphological information. This shared character probably indicates a close sister relationship between *B*. *ornatus* and the remaining four species; however, *B*. *ornatus* shows two distinct features: the absence of setal armature on the P2 exp-2 and P4 enp-2. We suspect that *B*. *ornatus* branches off near *B*. *clarae*.

There is no uncertainty regarding the monophyletic relationship of the three new species described herein, as shown in our cladograms (Figs. 21A, 21B) with a high bootstrap value (95%). These species share five synapomorphies: the maxillulary exopod is represented by two setae (character no. 25: 0); all exopodal segments of the P1 are ornamented with outer spinules (character no. 30: 0); the P2–P4 coxae have a group of setules (character no. 33: 0); the female P5 exopod has a tube pore (character no. 38: 0); and the male P5 baseoendopod has a tube pore next to the endopodal element (character no. 40: 1). In this clade, *B*. *huysi* sp. nov. shows a sister relationship with the *B*. *lacuna* sp. nov./*B*. *comptus* sp. nov. and represents three autapomorphies: the cephalothorax has additional protuberances between mediolateral extensions and posterolateral corniform processes (character no. 10: 1); the length of caudal rami is distinctly shorter than those of congeners (character no. 16: 1); and the outer element on the P4 enp-2 is short and delicate (character no. 36: 1). One autapomorphy, character no. 16, is also evident in *B*. *hamatus*, which was excluded from our cladistic analysis because of the rudimentary original description (see Thomson, 1883: Pl. X, fig. 22). The reduction of caudal rami length is presumed here to result from secondary evolution. Although George, Glatzel & Schröder (2019) suggested that *B*. *hamatus* initially branched from a hypothetical common ancestor, we suspect that this species has a close relationship to *B*. *huysi* sp. nov. To clarify their phylogenetic relationship, re-examination of *B*. *hamatus* and *B*. *ornatus* is urgently needed.

The three Korean species herein are considered distinct because of the autapomorphies defined in our cladistic analysis (see above). They can also be distinguished by the character state of the rostrum, cephalothoracic processes, female genital double-somite, caudal rami, and sexual dimorphic features of the male P3 enp-2.

A diagnostic character of the family Ancorabolidae is a noble apophysis on the P3 enp-2 in the male (George, 2020). This might be a shred of significant evidence to reflect a close relationship between the superfamilies Laophontoidea Scott T., 1904, and Cletodoidea Bowman & Abele, 1982. Based on the ontogeny of *Orthopsyllus* sp. (the family Orthopsyllidae Huys, 1990) in both sexes, Huys (1990) suggested that this apophysis in the male is derived from the outer spine on the second segment in the female with a secondary segmentation of the third segment (see Huys, 1990: Fig. 16). Even though the loss of this outer element in the females is expressed in most ancorabolid copepods, *Calypsophontodes macropodia* (Gee & Fleeger, 1986) retained the plesiomorphic condition that the outer element is present in the female P3 enp-2 (Gheerardyn & Lee, 2012). It leads us to postulate that the gene of the outer element repressed in the female is probably expressed in the male and modified into an apophysis. On the other hand, the males of *B*. *lacuna* sp. nov. exhibit a recurved tube pore in where the sexual dimorphic apophysis is present in other ancorabolid copepods. A similar phenotype is evident in the distal armature of the free antennary endopodal segment in *B*. *horstgeorgei*. A long tube pore is present in the position of a small seta found in *B*. *bicornis*, *B*. *lacuna* sp. nov., *B*. *comptus* sp. nov., and *B*. *huysi* sp. nov. In the case of the subfamily Danielsseniinae Huys & Gee in Huys et al., 1996 (Pseudotachidiidae Lang, 1936), the modification of setae into tubular structures or sensory aesthetascs can be observed in the mouth appendages (cf., Huys & Gee, 1993). Accordingly, we presume that the recurved tube pore in the male P3 enp-2 of *B*. *lacuna* sp. nov. is homologous to the apophysis on the same segment of other species of *Bicorniphontodes*.

## Conclusions

The present taxonomical study of harpacticoid copepods revealed high species diversity of *Bicorniphontodes* species in Korean waters, based on the discovery of three new species: *B*. *lacuna* sp. nov., *B*. *comptus* sp. nov., and *B*. *huysi* sp. nov. Following a careful reevaluation of this species based on materials from Udo Islet near Jeju Island, the previously reported *L*. *bicornis sensu* Kim, 2013 was revised to a distinct species (*B*. *huysi* sp. nov.). We attempted to reconstruct possible phylogenetic relationships through morphology analysis of six *Bicorniphontodes* species, with an outgroup taxon, *L*. *typicus*. Our findings suggest a monophyletic status shared among three new Korean species in the terminal node. To clarify the phylogenetic relationship of *Bicorniphontodes* species, there is a need to reexamine the limited taxonomic information regarding *B*. *hamatus* and *B*. *ornatus*.

## Additional Information and Declarations

### Field Study Permissions

The following information was supplied relating to field study approvals (*i.e*., approving body and any reference numbers): Fieldworks were approved by the Korea Institute of Ocean Science and Technology (KIOST) (project numbers: PE99913 and PG51650).

### Data Availability

The following information was supplied regarding data availability: The raw data are line drawings of three new species and the morphological character status for cladistic analysis.

### New Species Registration

The following information was supplied regarding the registration of a newly described species: Publication LSID: urn:lsid:zoobank.org:pub:CA40EAE8-2B46-40A1-88D5-B32A80ADDD05 *Bicorniphontodes lacuna*: urn:lsid:zoobank.org:act:CEC926FC-DC5C-4E2A-82E2-A27DEEC37173 *Bicorniphontodes comptus*: urn:lsid:zoobank.org:act:A35DF79E-6E51-49EB-9200-996ECE8F09F9 *Bicorniphontodes huysi*: urn:lsid:zoobank.org:act:C2AF1702-965F-41EC-8AAD-BC54F13A69AE.
